# High‐Efficiency TADF Dendritic Emitters Enabled by Synchronously Inhibiting Degenerated Triplet Excited States and Structural Relaxation Toward Solution‐Processed OLEDs with EQE Over 33%

**DOI:** 10.1002/advs.202524183

**Published:** 2026-01-18

**Authors:** Xiaoxiang Yu, Wei Ping, Chengshuang Song, Jinyang Zhao, Lei Hua, Junjie Wang, Shian Ying, Yuchao Liu, Zhongjie Ren, Shouke Yan

**Affiliations:** ^1^ Department Key Laboratory of Rubber‐Plastics School of Polymer Science and Engineering/State Key Laboratory of Advanced Optical Polymer and Manufacturing Technology Ministry of Education/Shandong Provincial Key Laboratory of Rubber‐Plastics Qingdao University of Science & Technology Qingdao P. R. China; ^2^ State Key Laboratory of Chemical Resource Engineering College of Materials Science and Engineering Beijing University of Chemical Technology Beijing P. R. China; ^3^ School of Materials Science & Engineering Changzhou University Changzhou P. R. China

**Keywords:** asymmetrical architecture, isolated triplet states, solution‐processed OLEDs, symmetry breaking, TADF dendrimers

## Abstract

Manufacturing high‐performance solution‐processed organic light‐emitting diodes (OLEDs) employing thermally activated delayed fluorescence (TADF) dendritic emitters remains a formidable challenge due to the lack of efficient TADF dendrimers. Herein, a symmetry breaking strategy is adopted to construct an asymmetrical TADF dendrimer denoted as **DMAC‐XT‐TCz**. An in‐depth analysis of the photophysical properties combining with theoretical calculation expose that the asymmetrical architecture of target emitter switches degenerated triplet excited states to isolated counterpart, thereby effectively breaking the degeneracy of vibrational levels and boosting the spin flip of triplet excitons. Additionally, the nonradiative decay is also suppressed due to imbedding oxygen linkage to locking electron‐donating skeleton. Thus, near‐unity photoluminescence efficiency and excellent reverse intersystem crossing rate of 7.8 × 10^5^ s^−1^ can be achieved for **DMAC‐XT‐TCz**. Impressively, the optimized solution‐processed OLEDs achieve an attractive external quantum efficiency of 33.7%, which is the highest value for TADF dendrimer‐based OLEDs. By using **DMAC‐XT‐TCz** as sensitizer, the solution‐processed narrowband OLEDs based on a multiple‐resonance TADF emitter also acquire record‐high device performances with current efficiency of 117.7 cd A^−1^. This study highlights the significance of asymmetric architecture in designing high‐efficiency TADF dendrimer, and provides an effective strategy to boost solution‐processed narrowband OLEDs through adopting TADF dendrimer as sensitizer.

## Graphical abstract

Asymmetrical thermally activated delated fluorescence dendritic emitters are delicately constructed through symmetry breaking strategy, and near‐unity photoluminescence efficiency and excellent reverse intersystem crossing process are achieved via breaking the degeneracy of vibrational levels and boosting the spin flip of triplet excitons. The optimized solution‐processed organic light emitting dioses achieve a record‐high external quantum efficiency of 33.7%.

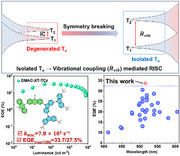

## 1 Introduction

Since thermally activated delayed fluorescence (TADF) mechanism was innovatively applied to explore advanced organic light‐emitting diodes (OLEDs), the designing principles of TADF‐based compounds have speedily evolved [1, 2, 3, 4, 5]. Most OLEDs panels are fabricated by precisely depositing manifold functional layers in sequence under ultra‐high vacuum, and numerous attractive TADF‐based OLEDs have emerged with emission ranging from deep‐blue to near‐infrared wavelengths [6, 7, 8, 9, 10, 11, 12, 13, 14]. Although the vacuum‐deposited OLEDs based on TADF small molecules have almost satisfied the request of industrialization in terms of device efficiency and stability, the predicament in fabricating large‐area flexible display equipment and excessively high fabrication cost during successive multi‐layer forming processes detract features of the vacuum‐deposited technology [15, 16]. As an alternative, solution‐processing manufacture can also fabricate OLEDs via low‐cost spin‐coating or inkjet printing technique by harnessing high‐performance polymeric or dendritic TADF emitters due to excellent film‐forming ability, and requisite thermal and morphological stability [17, 18, 19, 20, 21, 22, 23, 24].

Solution‐processed OLEDs can be produced using elaborately designed light‐emitting polymers, which are endowed with charge‐transporting and light‐emitting capacities simultaneously in their polymer chain [25, 26]. Nevertheless, the OLEDs based polymeric emitters have limited device efficiency, which has impeded their commercial use. From the perspective of molecular design, polymeric emitters possess inevitable defects induced by unreacted active groups, photo‐generated free radicals, chain entanglements and inter–chain interactions [27, 28, 29, 30, 31], which also bring about inherently inferior repeatability of the photoelectric properties for each batch. In contrast, dendritic emitters have accurate chemical structure and defined molecular weight, and the bulky molecular structures of dendrimers with large steric hindrance can enlarge intermolecular distance, which can effectively mitigate aggregation caused quenching (ACQ) effect [13, 32, 33]. Additionally, the energy splitting between singlet and triplet excited states (Δ*E*_ST_) and exciton decay dynamics behavior can be reasonably modulated by modular molecular design using dendronized donors or acceptors [20, 34, 35, 36]. However, the device performance for solution‐processed OLEDs employing dendritic TADF emitters still lags far behind the vacuum‐deposited counterparts, hence high‐efficiency dendritic TADF emitters are still highly desired.

The improvement of device performance intensively depends on optimizing reverse intersystem crossing (RISC) channel for TADF dendrimers, so the rate of reverse intersystem crossing (*k*_RISC_) becomes the key factor to realize high external quantum efficiency (EQE) values [37, 38]. According to Fermi's Golden rule, enhancing spin‐orbital coupling (SOC) strength between singlet and triplet excited state is a practical strategy to facilitate RISC [39]. Asymmetric architecture has been proven to accelerate the spin flip of triplet excitons via promoting mixing of multiple triplet excited states, in which the high‐lying excited states with different spin multiplicities and transition dramatically accelerate RISC process due to enhanced SOC strength [40, 41, 42]. Moreover, asymmetric TADF dendrimers can effectively prevent deterioration of electron coupling coefficient between electron‐donating dendron and acceptor core and the oscillator strength (*f*) when integrating an auxiliary dendron into dendrimer, which is regarded as inevitable in symmetric dendrimers [17, 43, 44]. In previous work, we proposed an asymmetric TADF dendrimer (**DMAC‐BP‐*t*Bu3Cz**) by integrating carbazole as peripheral dendrons with donor−acceptor (D‐A) type molecule of 4‐(9,9‐dimethyl‐9,10‐dihydroacridine)‐benzophenone [40], achieving instrumentally high SOC values and correspondingly *k*_RISC_ of exceeding 10^6^ s^−1^. However, the root mean squared displacement (RMSD) values between ground and emissive states of these TADF dendrimers are much higher than that of conventional fluorescent dyes with locally excited (LE) dominated excited states, resulting in intrinsically lower radiative decay rates (*k*_r_s) than those of LE‐dominated emitters, and thus severe exciton losses especially singlet‐singlet annihilation (SSA) and singlet‐triplet annihilation (STA) are prevalent in TADF dendrimers [9, 45, 46]. In this regard, the high‐efficiency TADF dendrimers should be designed with caution to boost SOC strength and meanwhile retain enough small RMSD values.

**FIGURE 1:**
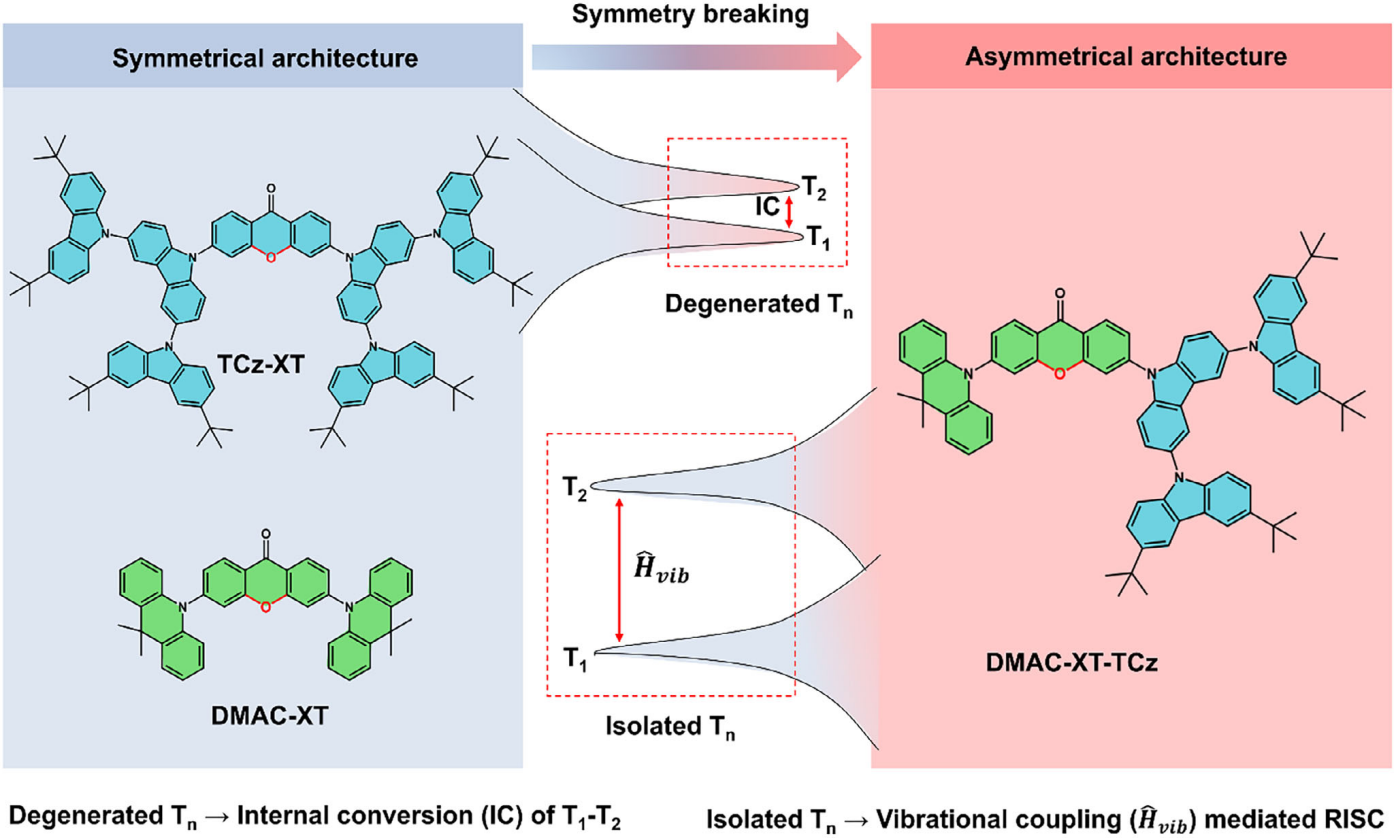
Molecular structures of newly‐designed symmetrical emitters of **TCz‐XT** and **DMAC‐XT**, and asymmetrical emitter of **DMAC‐XT‐TCz**. Schematic diagram of triplet energy level (*T*_n_) distribution for symmetrical and asymmetrical emitters.

Herein, a symmetry breaking strategy is adopted to construct an asymmetrical dendritic TADF emitter denoted as **DMAC‐XT‐TCz**. Then two symmetrical TADF molecules named **TCz‐XT** and **DMAC‐XT** as control are also synthesized. This strategy involves the deliberate excited‐state modulation. As illustrated in Figure 1, two symmetrical molecules feature degenerated triplet excited states (*T*_n_), which have been demonstrated to detrimental exciton loss through intramolecular interaction. Therefore, the triplet excitons would be partly wasted in internal conversion (IC) processes, eroding photoluminescence efficiency and device performance. On the contrary, the asymmetrical architecture of tailored TADF emitter, **DMAC‐XT‐TCz**, switches degenerated *T*_n_ to isolated counterpart, thereby effectively breaking the degeneracy of the vibrational level. Moreover, the appropriate energy‐level difference between T_1_ and T_2_ evokes favorable vibronic coupling (${\hat{H}}_{\mathrm{vib}}$), which serves as a mediator to enhance RISC process through promoting the mixing of multiple triplet excited states to boost SOC interaction between triplet and singlet. Thus, the calculated SOC matrix element of **DMAC‐XT‐TCz** is relatively higher than that of **TCz‐XT** and **DMAC‐XT**, which will hasten the spin flip of triplet excitons to emissive states in asymmetric **DMAC‐XT‐TCz**. As a result, near‐unity photoluminescence efficiency and excellent *k*_RISC_ of 7.8 × 10^5^ s^−1^ can be achieved for **DMAC‐XT‐TCz**. Impressively, the optimized solution‐processed OLEDs achieve an attractive EQE of 33.7%, which is highest value for TADF dendrimer based OLEDs. By using **DMAC‐XT‐TCz** as sensitizer, the solution‐processed narrowband OLEDs based on a multiple‐resonance TADF emitter (**TCz‐BN**) acquire dramatically improved device performances with EQE_max_ of 30.2% and record‐high current efficiency of 117.7 cd A^−1^. This study highlights the significance of asymmetric architecture in designing high‐efficiency TADF dendrimer, and provides an effective strategy to boosts solution‐processed narrowband OLEDs through adopting TADF dendrimer as sensitizer. Our strategy accomplishes the boosted luminescence efficiency of MR‐TADF emitters through enabling the participation of multiple triplet states and the confined excited‐state conformations induced by intermolecular CT interaction in aggregation state.

## 2 Results and Discussion

### 2.1 Molecular Design and Theoretical Investigation

The molecular structures of newly‐designed emitters are shown in Figure 1a, in which symmetric compounds named **TCz‐XT** and **DMAC‐XT** separately adopt 3,3'',6,6''‐tetra‐tert‐butyl‐9'H‐9,3':6',9''‐tercarbazole (TCz) and 9,9‐dimethyl‐9,10‐dihydro‐acridine (DMAC) as the electron‐donating moieties while ring‐closed xanthenone (XT) as an electron‐withdrawing unit. As for the asymmetric dendritic emitter **DMAC‐XT‐TCz**, DMAC and second‐generation TCz dendron were together integrated with XT acceptor. The detailed synthetic routes of these emitters are exhibited in Schemes S1−S7, and the corresponding chemical structures are confirmed by ^1^H NMR, ^13^C NMR, and MS spectra (Figures S1−S19). All three emitters exhibit high thermal stability, as evidenced by a high decomposition temperature (*T*_d_) of 370°C–476°C at a 5% loss of the initial weight from thermogravimetric analyses (Figure S20).

**FIGURE 2:**
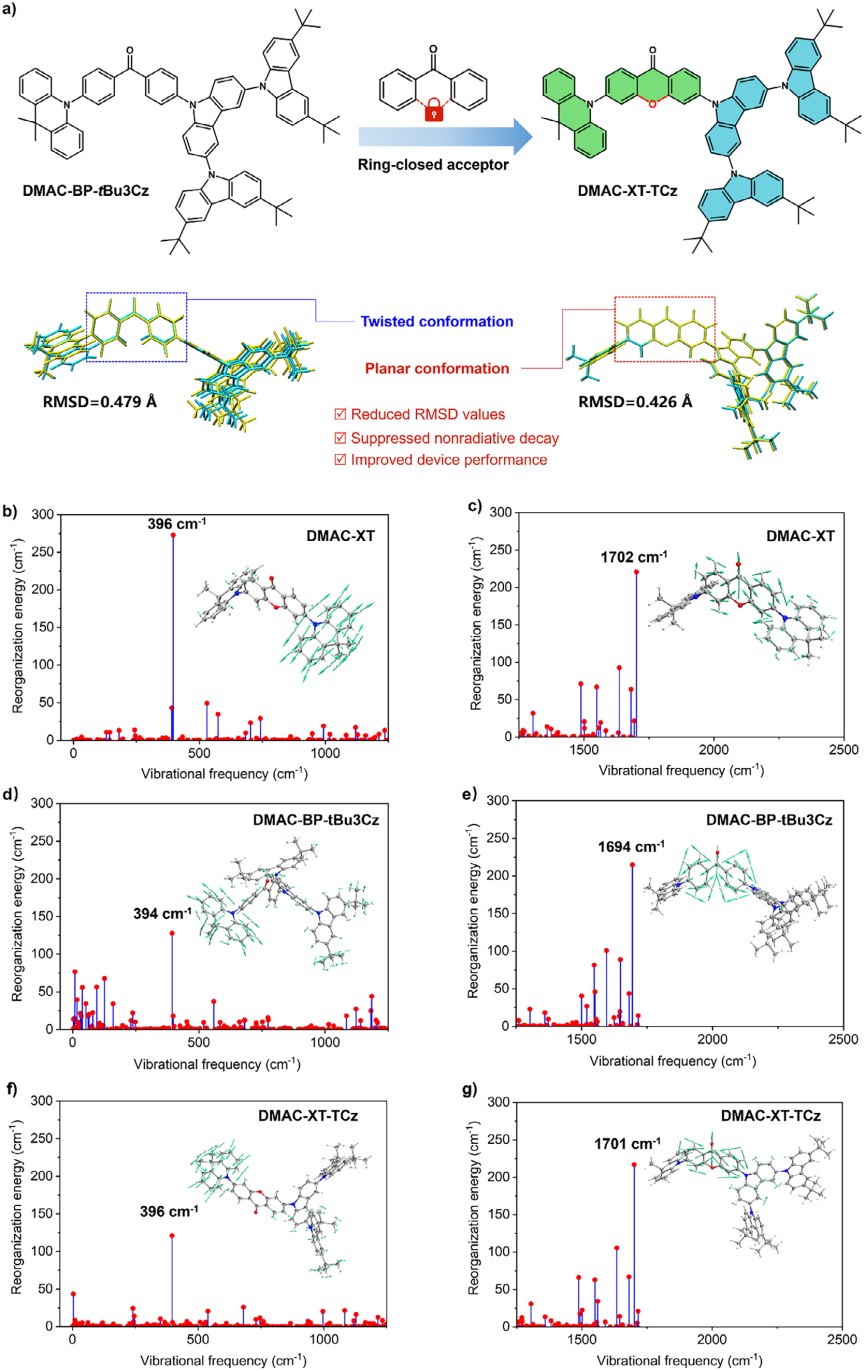
(a) Schematic illustration of molecular design strategy of ring‐closed acceptor. Calculated root mean squared displacement (RMSD) between optimized S_0_ (yellow) and S_1_ (blue) geometries for **DMAC‐BP‐tBu3Cz** and **DMAC‐XT‐TCz**. Reorganization energy versus the vibrational frequency of (b) (c) **DMAC‐XT**, (d) (e) **DMAC‐BP‐tBu3Cz**, and (f) (g) **DMAC‐XT‐TCz** (inset: most significant vibration modes).

Theoretical calculations based on density functional theory (DFT) have been initially conducted to elucidate the relevance of the molecular architectures to their SOC strength and RMSD values. As depicted in Figure S21, asymmetrical compounds possess relatively higher SOC matrix element ($\left\langle S_{1}|{\hat{H}}_{\mathrm{SOC}}|T_{n}\right\rangle$ n = 1, 2) than the corresponding symmetrical emitters, proving that asymmetrical architecture can boost SOC strength between singlet and triplet excited states, which is expected to accelerate spin flip of triplet excitons. In addition, structural changes during the lowest singlet excited state (S_1_) → ground state (S_0_) transitions could be deliberately evaluated using RMSD values. As anticipated, the previously reported emitters adopting benzophenone skeleton as electron‐withdrawing moiety exhibit relatively larger RMSD values which originate from the fluctuation of the flexible framework (Figure 2a; Figure S22). Concretely, the RMSD values of **DMAC‐XT‐TCz** (0.426 Å) is lower than that of **DMAC‐BP‐*t*Bu3Cz** (0.471 Å) because of a rigid polycyclic structure of planar XT skeleton, which is constructed by ring closure with an oxygen linkage in twisted benzophenone framework, and thus nonradiative decay can be suppressed. Although the XT unit with a closed‐loop structure formed by oxygen bridge linkage can indeed improve the molecular planarity and reduce the RMSD value. However, the tert‐butyl structure of the TCz unit introduced into the dendritic moiety results in a less significant reduction in the RMSD value of **DMAC‐XT‐TCz** compared with that of **DMAC‐XT** (Figure S21). Moreover, quantitative analysis of the intramolecular motions of **DMAC‐XT**, **DMAC‐BP‐*t*Bu3Cz**, and **DMAC‐XT‐TCz** has been conducted through reorganization energy calculation. The principal vibrational modes of three emitters are identified at frequencies of 394–396 cm^−1^ in the range of 0–1200 cm^−1^, primarily arising from the twisting and rotation vibration of the electron‐donating units of DMAC groups and peripheral dendrons, and the vibrational mode diagrams are depicted in Figure 2b–g. It can also be easily detected that the high‐frequency (1200–2000 cm^−1^) modes are associated with the vibrations localized primarily on the electron‐withdrawing units of XT or BP groups. Both **DMAC‐BP‐*t*Bu3Cz** and **DMAC‐XT‐TCz** exhibit relatively smaller reorganization energies of 127.4 and 120.8 cm^−1^ than **DMAC‐XT** (272.4 cm^−1^) despite **DMAC‐XT** features rigid molecular skeleton, indicating that asymmetrical architecture potentially enables limited intramolecular motions for electron‐donating groups. Notably, vibration modes at frequencies of 0–200 cm^−1^ are significantly restrained when incorporating XT group into emitters, further demonstrating that rigid polycyclic structure of XT skeleton decrease the number of vibration modes. Surprisingly, three emitters exhibit nearly consistent vibrational modes with primary frequencies of 1694–1702 cm^−1^, accompanying by extremely close reorganization energies of around 215 cm^−1^. Although the suppressed high‐frequency modes are inconspicuous in emitters based on XT acceptor, the favorable changes on low‐frequency modes for **DMAC‐XT‐TCz** potentially enable the crucial suppression of non‐radiative energy lose and gaining attractive PL QY values. Additionally, the reorganization energy values of the three emitters were also evaluated via the four‐point method, 0.57 eV for **TCz‐XT**, 0.29 eV for **DMAC‐XT**, and 0.32 eV for **DMAC‐XT‐TCz**, respectively.

The optimized structures and corresponding energy levels of fragments (containing XT, DMAC and TCz) were also evaluated. As depicted in Figure S23, the lowest unoccupied molecular orbital (LUMO) energy level of the XT moiety is −1.68 eV, while the highest occupied molecular orbital (HOMO) energy levels of the DMAC and TCz moiety are −5.13 and −5.22 eV, respectively, indicating that the intramolecular charge transfer (ICT) from DMAC to XT fragments is decisive to the photophysical properties for dendritic emitter of **DMAC‐XT‐TCz**. Then the HOMO and LUMO values are also determinated to be −5.32/−2.20 eV for **TCz‐XT**, −5.30/−2.07 eV for **DMAC‐XT** (Figure S24). While for **DMAC‐XT‐TCz**, the HOMO/HOMO‐1 are localized on TCz and DMAC moieties, respectively. Additionally, the orbital contribution of HOMO‐1→LUMO to the S_1_ state was determined to be 95.2%. The energy levels of HOMO‐1/LUMO are −5.34/−2.14 eV for **DMAC‐XT‐TCz**.

### 2.2 Steady‐State and Transient Photophysical Properties

**FIGURE 3:**
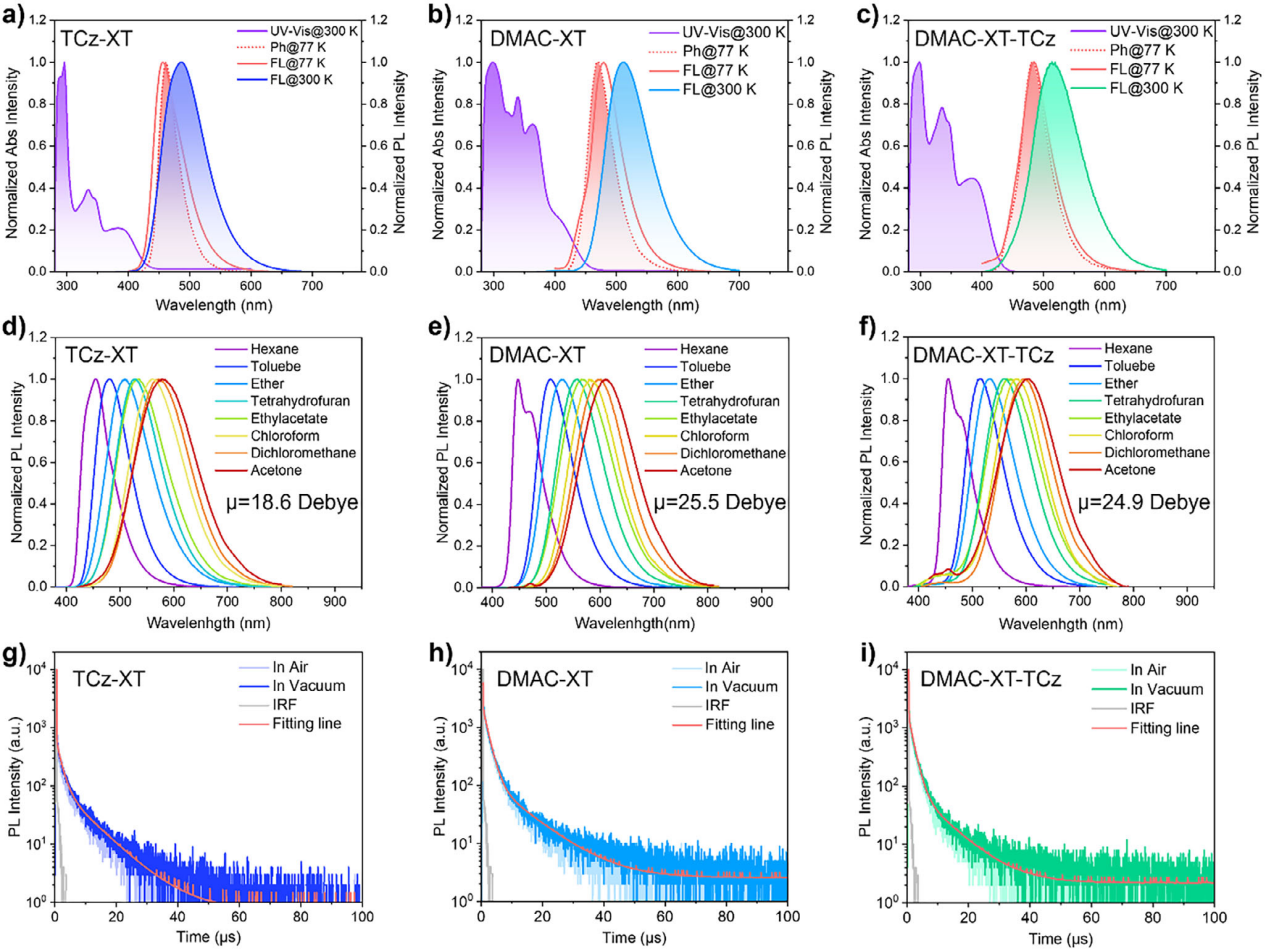
Steady‐state and transient photophysical properties of three emitters. UV−vis absorption and fluorescence spectra at 77 and 300 K, and phosphorescence spectra at 77 K of (a) **TCz‐XT**, (b) **DMAC‐XT**, and (c) **DMAC‐XT‐TCz**. Solvatochromic PL spectra of (d) **TCz‐XT**, (e) **DMAC‐XT**, and (f) **DMAC‐XT‐TCz** in diluted solution (10^−5^ m). Transient PL decay characteristics of (g) **TCz‐XT**, (h) **DMAC‐XT**, and (i) **DMAC‐XT‐TCz** based doped films in air and vacuum at 300 K.

To evaluate the photophysical properties of monomolecular species, the UV–vis absorption and photoluminescence (PL) spectra of three emitters diluted in toluene were initially performed as presented in Figure 3a–c. Three emitters exhibit major absorption bands at around 300 nm, which should be ascribed to the localized π–π* transitions of the conjugated skeletons. The UV–vis absorption spectra also confirm the presence of prominent charge transfer (CT) transition bands ranging from 330 to 450 nm. According to onset of the bands, the energy gaps (*E*_g_s) between HOMO and LUMO energy levels can be determined to be 2.85, 2.69, and 2.66 eV for **TCz‐XT**, **DMAC‐XT**, and **DMAC‐XT‐TCz**, respectively. Combining with HOMO values from cyclic voltammetry results (−5.55 eV for **TCz‐XT**, −5.34 eV for **DMAC‐XT**, and −5.55 eV for **DMAC‐XT‐TCz**) shown in Figure S26, the LUMO values can be computationally determined to be −2.70, −2.65, and −2.89 eV for **TCz‐XT**, **DMAC‐XT**, and **DMAC‐XT‐TCz**, respectively.

From the PL spectra, the maximum emission peaks are 486, 510, and 515 nm for **TCz‐XT**, **DMAC‐XT**, and **DMAC‐XT‐TCz**, respectively. Notably, the PL spectrum of **DMAC‐XT** is dramatically redshifted by 24 nm compared with that of **TCz‐XT**, signifying that the LUMO level of TCz moiety is deeper than that of DMAC moiety, which is in accordance with the DFT calculated energy levels. Due to the relatively shallower LUMO level of DMAC fragment, the dominated photophysical process of asymmetric **DMAC‐XT‐TCz** stems from intramolecular CT transition from DMAC to XT moieties. As the tiny Δ*E*_ST_ value is a prerequisite for accelerating upconversion of triplet excitons, the Δ*E*_ST_ values of three emitters were then calculated by estimating the energies of S_1_ and triplet (T_1_) excited states from the onset values of fluorescence and phosphorescence spectra in toluene at 77 K. Excitedly, asymmetric **DMAC‐XT‐TCz** possesses Δ*E*_ST_ of only 18 meV, which is extremely smaller than that of **TCz‐XT** (68 meV) and **DMAC‐XT** (51 meV), potentially ensuring the establishment of more effective channels for the triplet‐to‐singlet RISC process in **DMAC‐XT‐TCz**.

According to the solvatochromic effects depicted in Figure 3d–f, the emissive excited states of three emitters were assessed. All three emitters exhibit apparent solvatochromic effects, revealing that the dominant emissive excited states feature CT characteristics. In low‐polar hexane solvents, **DMAC‐XT** and **DMAC‐XT‐TCz** exhibit similar emission profiles with maximum peaks at around 450 nm along with an extra high‐energy band at around 470 nm while **TCz‐XT** displays a single emission band. When increasing the solvent polarity from hexane to acetone, the emission spectra of **TCz‐XT** reveal moderate bathochromic shift of 125 nm. Comparatively, the solvatochromism of emission peak for both **DMAC‐XT‐TCz** and **DMAC‐XT** is more conspicuous with bathochromic shift of 150–160 nm when altering solvents from hexane to acetone, demonstrating much higher dipole moment (µ) values of 25.5 and 24.9 Debye for **DMAC‐XT** and **DMAC‐XT‐TCz** than that of 18.6 Debye for **TCz‐XT** (Figure S27). Notably, the close µ values between **DMAC‐XT** and **DMAC‐XT‐TCz** further prove that the photophysical process of asymmetric **DMAC‐XT‐TCz** are dominated by intramolecular CT transition from DMAC to XT moieties.

The PL decay curves of the doped films (10 wt.% emitters blended with 9‐(3‐(9H‐carbazol‐9‐yl) phenyl)‐9H‐carbazole‐3‐carbonitrile (mCPCN) as host materials) were also prudently detected in vacuum and air conditions. As shown in Figure 3g–i, all decays feature biexponential decay, consisting of prompt fluorescence (PF) regimes and subsequently delayed fluorescence (DF) components. Hence, triplet excitons have been proven to be involved in photophysical process with the DF component proportions (φ_DF_s) of 30.4%, 49.9% and 58.8%, for **TCz‐XT**, **DMAC‐XT**, and **DMAC‐XT‐TCz** blended films, respectively. Moreover, in good consistence with the relatively small Δ*E*_ST_ and high SOC strength between singlet and triplet excited states, **DMAC‐XT‐TCz** reveals greatly more efficient exciton decay kinetic characteristic with shorter DF lifetime (τ_d_) of 3.0 µs and faster *k*_RISC_ of 7.8 × 10^5^ s^−1^ than **TCz‐XT** (τ_d_ = 5.2 µs, *k*_RISC =_ 2.4×10^5^ s^−1^) and **DMAC‐XT** (τ_d_ = 4.1 µs, *k*_RISC =_ 4.8×10^5^ s^−1^), manifesting that exciton spin‐flip processes are efficaciously accelerated via constructing asymmetric architecture in **DMAC‐XT‐TCz**. Therefore, the photoluminescence quantum yield (Φ_PL_) values of blended films can reach 95% for **DMAC‐XT‐TCz**. Additionally, the PF lifetimes (τ_p_s) were determined to be 26.4, 24.5, and 24.3 ns for **TCz‐XT**, **DMAC‐XT**, and **DMAC‐XT‐TCz**, respectively, and thus the *k*_r_s could be calculated to be 1.7–2.6×10^7^ s^−1^ for three emitters, which are apparently higher than those of benzophenone based dendrimers with high RMSD values (< 10^7^ s^−1^) [40, 47, 48]. Moreover, the nonradiative decay rates ($k_{\mathrm{nr}}^{s}$) of **DMAC‐XT‐TCz** is determined to be 0.8 × 10^6^ s^−1^, which is significantly inhibited compared with **TCz‐XT** (4.9 × 10^6^ s^−1^) and **DMAC‐XT** (1.6 × 10^6^ s^−1^). All these photophysical properties are summarized in Table 1. These considerations suggest that a rigid electron‐withdrawing fragments with asymmetric molecular architectures can enable boosted *k*_RISC_ and *k*_r_ values and suppressed $k_{\mathrm{nr}}^{s}$ simultaneously for TADF dendritic emitters, which are pivotal to explore robust solution‐processed OLEDs.

**TABLE 1:** The photophysical properties of emitters.

Emitter	*λ*_abs_ [nm]^a^	*λ*_PL_ [nm]^b^	Δ*E*_ST_ [meV]^c^	Φ_PL_ [%]^d^	τ_p_/τ_d_ [ns]/[µs]^e^	φ_DF_ [%]^f^	*k*_r_ [10^7^ s^−1^]^g^	*k*_RISC_ [10^5^ s^−1^]^h^	$k_{nr}^{S}$ [10^6^ s^−1^]^i^
TCz‐XT	296, 335	486	68	87	26.4/5.2	30.4	2.6	2.4	4.9
DMAC‐XT	298, 339	510	51	98	24.5/4.1	49.9	2.0	4.8	1.6
DMAC‐XT‐TCz	304, 353	515	18	95	24.3/3.0	58.8	1.7	7.8	0.8

^a^ absorption peaks of emitters diluted in toluene.

^b^ maximum emission peaks of emitters in diluted in toluene.

^c^ energy splitting between S_1_ and T_1_ determinated from diluted solutions.

^d^ photoluminescence quantum yields of doped films.

^e^ the lifetimes of prompt fluorescence component and the lifetimes of delayed fluorescence component.

^f^ the proportion of delayed fluorescence components.

^g^ the rate constants of radiative decay by equation of *k_r_* = (1 − φ_DF_)/τ_p_.

^h^ the rate constants of reverse intersystem crossing process calculated by equation of *k*_RISC_ = ∅_PL_/[τ_d_(1 − φ_DF_)].

^i^ the rate constants of nonradiative decay for triplet excitons id given by equation of $k_{\mathrm{nr}}^{S}=\left(1-\emptyset_{\mathrm{PL}}\right)/\tau_{p}$.

### 2.3 Excited‐State Calculation and Analysis

**FIGURE 4:**
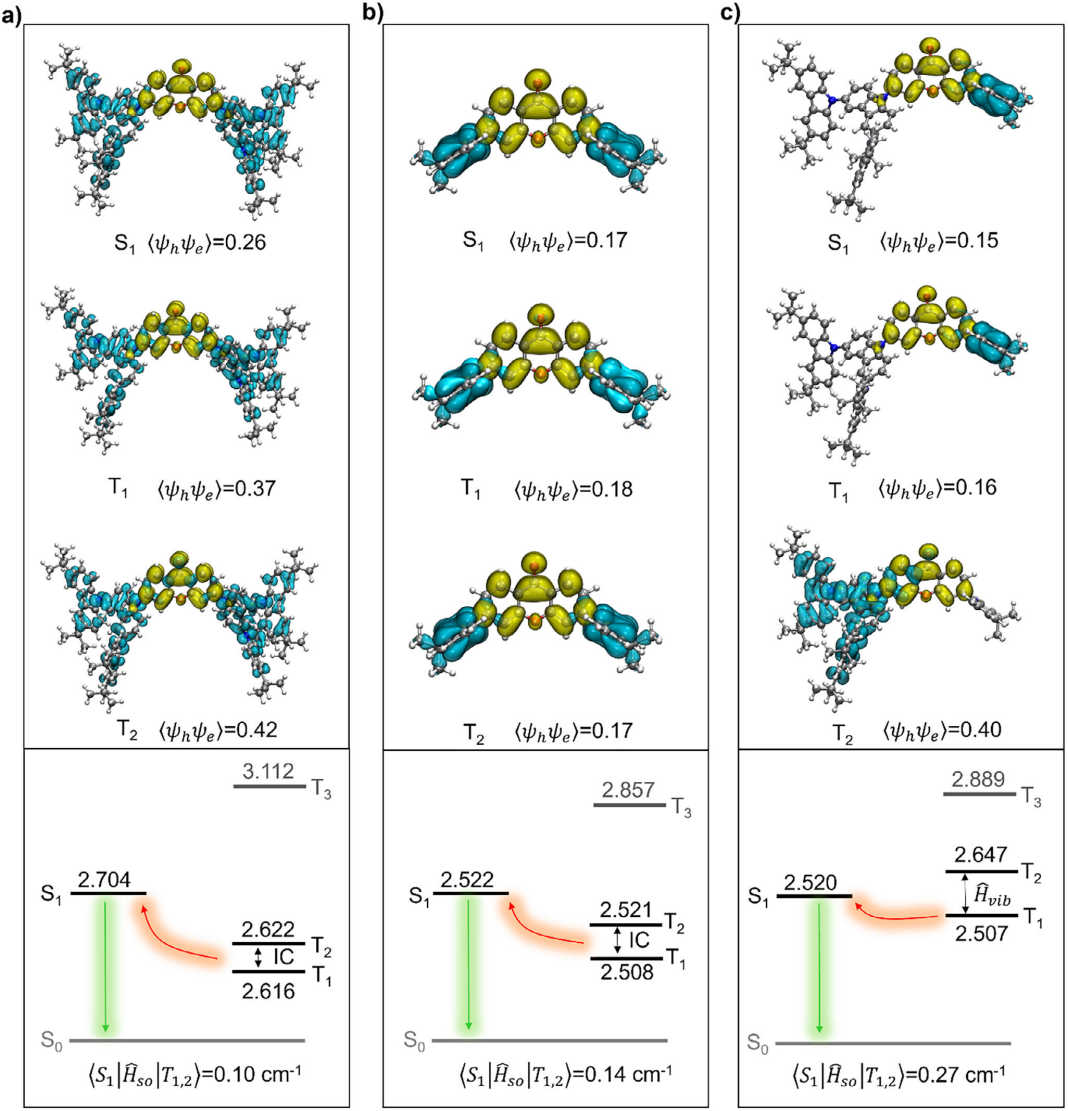
Natural transition orbitals of *S*_1_ and *T*_n_ (n = 1,2) states, and the schematic energy diagram of the related excited states for (a) **TCz‐XT**, (b) **DMAC‐XT**, and (c) **DMAC‐XT‐TCz**, respectively. The blue and yellow regions represent hole and particle distributions, respectively.

To further understand the nature of excited states, natural transition orbital (NTO) analyses were performed for the S_1_ and *T*_n_ (n = 1, 2) excited states of three emitters based on optimized molecular structures [49, 50]. The systematic benchmarking of different functions was also conducted for excitation/emission energy calculations by using **DMAC‐XT‐TCz** as emitter, and then PBE1PBE/6‐31G(d) method was judiciously adopted (Table S1 and Figure S25). As depicted in Figure 4, the NTO distributions of the S_1_ states are quite similar for three emitters, where the particles are mainly distributed on the electron‐withdrawing XT moiety while holes are concentrated on electron‐donating moieties, accompanied by a relatively small overlap integral (〈ψ_h_ψ_e_〉), indicating convinced CT‐dominated transition natures. Notably, the hole distribution of the S_1_ states for **DMAC‐XT‐TCz** is in accord with that for **DMAC‐XT**, computationally verifying the predominant intramolecular CT transition from DMAC to XT moieties in asymmetric **DMAC‐XT‐TCz**. Additionally, the 〈ψ_h_ψ_e_〉 value of **TCz‐XT** (0.26) is relatively higher than that of **DMAC‐XT** (0.17) and **DMAC‐XT‐TCz** (0.15), which is consistent with the smaller µ value of **TCz‐XT** determined by solvatochromism mentioned above. For the *T*_n_ (n = 1, 2) states, the 〈ψ_h_ψ_e_〉 value of **TCz‐XT** increases to 0.37 (T_1_) and 0.42 (T_2_), indicating much more locally‐excited (LE) natures, while **DMAC‐XT** still maintains typically CT natures with almost same 〈ψ_h_ψ_e_〉 values of 0.18 (*T*_1_) and 0.17 (*T*_2_). Intriguingly, the *T*_2_ state of **DMAC‐XT‐TCz** demonstrates coexistence of LE and CT natures with 〈ψ_h_ψ_e_〉 of 0.40 although *T*_1_ state features CT characteristic with 〈ψ_h_ψ_e_〉 of 0.16. Different natures of singlet and triplet excited states enable boosted SOC strength according to the law of the conservation of angular momentum [51, 52]. Thus, the calculated SOC matrix element ($\left\langle S_{1}|{\hat{H}}_{so}|T_{1,2}\right\rangle$) between *S*_1_ and *T*_n_ (n = 1, 2) of **DMAC‐XT‐TCz** (0.27 cm^−1^) is relatively higher than that of **TCz‐XT** (0.10 cm^−1^) and **DMAC‐XT** (0.14 cm^−1^), which will hasten the spin flip of triplet excitons to emissive states in asymmetric **DMAC‐XT‐TCz**.

In the cases of energy levels, the calculated Δ*E*_ST_ value between *S*_1_ and *T*_1_ of **DMAC‐XT‐TCz** (13 meV) is fairly approximate to that obtained from photophysical analyses, which is also smaller than that of **TCz‐XT** (88 meV) and **DMAC‐XT** (14 meV). Moreover, the *T*_2_ energy levels of **TCz‐XT** and **DMAC‐XT** are almost equal to corresponding *T*_1_ energy levels with extremely tiny energy gaps between *T*_1_ and *T*_2_ state for **TCz‐XT** (6 meV) and **DMAC‐XT** (13 meV), potentially evoking conical intersections between *T*_2_ and *T*_1_ states, which will provoke detrimental IC process to compete with RISC process [9, 17, 53]. Nevertheless, the degenerated triplet excited states are appreciably suppressed for asymmetric **DMAC‐XT‐TCz** with 140 meV energy splitting between *T*_1_ and *T*_2_ sates, which makes it possible to enable favorable ${\hat{H}}_{\mathrm{vib}}$, and thus promoting the mixing of multiple triplet excited states to boost SOC interaction between triplet and singlet. Therefore, the attenuated degeneration of triplet excited states in asymmetric **DMAC‐XT‐TCz** can effectively restrain detrimental IC process and nonradiative decay, and assure a favorable spin‐flip process followed by a fast radiative transition. Notably, the T_3_ energy levels of three emitters lie much higher to their corresponding T_2_ energy levels, thoroughly restraining intrasystem interaction between T_2_ and T_3_ states. According to the Marcus equation, *k*_RISC_ is mainly related to three parameters including Δ*E*_ST_, SOC matrix element and recombination energy (λ). The calculated λ values are 0.57, 0.29, and 0.35 eV for **TCz‐XT**, **DMAC‐XT,** and **DMAC‐XT‐TCz**, respectively. Thus, the *k*_RISC_ values could be calculated to be 2.34 × 10^3^ s^−1^ (S_1_‐*T*_1,2_) for **TCz‐XT**, 5.64 × 10^5^ s^−1^ (S_1_‐T_1,2_) for **DMAC‐XT**, 5.67 × 10^4^ s^−1^ (S_1_‐T_1,2_, reformative value: 1.04 × 10^5^s^−1^) for **DMAC‐XT‐TCz**. It should be noted that the *k*_RISC_ value of **TCz‐XT** is extremely lower than that from photophysical analysis due to exaggerated λ value of TCz groups while the overlarge *k*_RISC_ value of **DMAC‐XT** can be attributed to the neglect of IC process between *T*_1_ and *T*_2_.

### 2.4 Electroluminescent Properties

**FIGURE 5:**
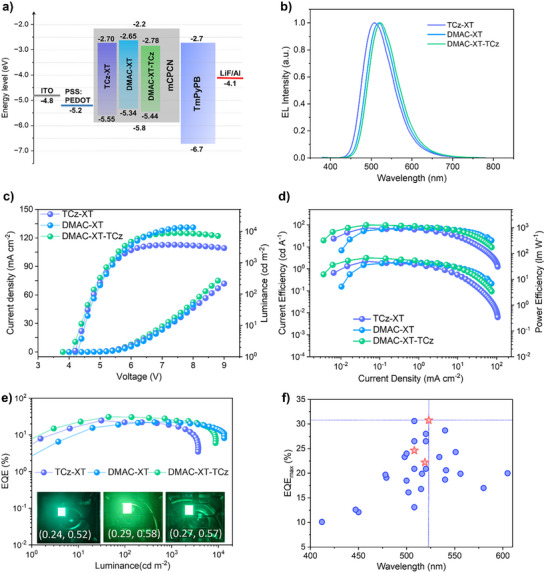
The EL performance of three newly‐designed emitters. (a) Energy diagram and the device architectures. (b) EL spectra at a voltage of 6 V for the devices. (c) Current density–voltage–luminance (*J–V–L*) curves. (d) The CE_max_ and PE_max_ as a function of current density. (e) EQE as a function of luminance. (f) Maximum EQE summary of the representative solution‐processed OLEDs based on TADF dendritic emitters with emission peak from 400 to 610 nm.

The morphology of the emitting film is critical for solution processed OLEDs. Therefore, the morphologies of the spin coated neat films of three dendritic emitters were initially studied using atomic force microscopy. As shown in Figure S28, the neat films have relatively smooth surface topographies with a root‐mean‐square (RMS) surface roughness of 0.267 nm for **TCz‐TX**, 0.258 nm for **DMAC‐TX** and 0.208 nm for **DMAC‐XT‐TCz**. The enough small RMS values especially for **DMAC‐XT‐TCz** can enable smooth and flat emitting layer even in blended films in high‐efficiency solution processed OLEDs. To verify the potential of these emitters in electroluminance (EL) devices, we fabricated OLEDs with multilayer configurations of indium tin oxide (ITO)/poly(3,4‐ ethylenedioxythiophene): polystyrene sulfonate (PEDOT: PSS, 30 nm, hole‐injecting layer)/emitting layer (30 nm)/1,3,5‐tri(m‐pyridin‐3‐ylphenyl)benzene (TmPyPB, 35 nm, electron transporting layer)/LiF(0.9 nm, electron‐injecting layer)/Al (110 nm) (Figure 5a). For the emitting layer, the **DMAC‐XT‐TCz**‐based devices with different doping concentrations under the same device architecture were initially tested as shown in Figure 5 and Figure S29, and eventually, and the dopant concentration was optimized to be 10 wt%. Eventually, 10 wt% emitters were embedded into host materials of mCPCN via solution blending to improve the carrier transferring balance and further restrain the aggregation caused quenching (ACQ) of excitons at high current density. The EL spectra at 6 V are shown in Figure 5b. Similar with the PL spectra, the emission band of **DMAC‐XT‐TCz** (516 nm) is quite similar to that of **DMAC‐XT** (520 nm), representing a bathochromic shift over 10 nm compared with **TCz‐XT** (504 nm). The coordinates of Commission Internationale de L'Eclairage (CIE) are (0.22, 0.50) for **TCz‐XT**, (0.28, 0.58) for **DMAC‐XT**, and (0.26, 0.56) for **DMAC‐XT‐TCz**, respectively. Although spectral redshifts in blended films are inevitable when employing high‐polar mCPCN as host material, the efficient triplet confinement and balanced carrier transporting abilities enable mCPCN to serve as widely used host material in OLEDs. From the current density–voltage–luminance (*J–V–L*) curves shown in Figure 5c, the turn‐on voltages (*V*_on_) of devices are 4.5–4.9 V, and the maximum luminance (*L*_max_) values are 4052, 9882, and 7784 cd m^−2^ for **TCz‐XT**, **DMAC‐XT**, and **DMAC‐XT‐TCz**, respectively. The maximum current (*CE*_max_) and power (*PE*_max_) efficiencies are 106.5 cd A^−1^ and 66.0 lm W^−1^ for **DMAC‐XT‐TCz**, which are dramatically higher than those of 71.1 cd A^−1^ and 37.6 lm W^−1^ for **TCz‐XT**, and 83.7 cd A^−1^ and 50.5 lm W^−1^ for **DMAC‐XT**, respectively (Figure 5d).

The relatively excellent device performance parameters of **DMAC‐XT‐TCz** are well consistent with its corresponding preponderant photophysical properties of superior PLQY (95%) and *k*_RISC_ (7.8 × 10^5^ s^−1^). Accordingly, the EQE_max_ value of **DMAC‐XT‐TCz** can reach 33.7% (Figure 5e), and the EQE can maintain relatively high values of 27.5% even at 1000 cd m^−2^, manifesting that SSA and STA can be effectively suppressed in asymmetric emitter due to enhanced SOC strength and decreased RMSD values for **DMAC‐XT‐TCz**. The photos of these OLED devices are also inserted in Figure 5e. As illustrated in Figurer 5f, to our best knowledge, the EQE value of 33.7% for **DMAC‐XT‐TCz** based device is the highest value for TADF dendrimer based solution‐processed OLEDs. The reported TADF dendrimer based solution‐processed OLEDs are summarized in Table S3. For comparison, the control solution‐processed OLEDs based on **DMAC‐XT** and **TCz‐XT** exhibit slightly lower EQE_max_ values of 25.6% and 24.9%, respectively, maintaining EQE values of 24.5% and 20.8% at 1000 cd m^−2^. All these device performance parameters are summarized in Table 2. The dramatically optimized device performance for **DMAC‐XT‐TCz** could be ascribed to the accelerated spin flip of triplet excitons and reduced nonradiative decay due to the favorably mixing of multiple triplet excited states and effectively prohibited degeneracy of the vibrational level induced from symmetry breaking in **DMAC‐XT‐TCz**.

**TABLE 2:** The electroluminescence properties of emitters.

Emitter	λ_EL_ [nm]^a^	FWHM [nm]^b^	V_on_ [V]^c^	L_max_ [cd m^−2^]^d^	CE_max_ [cd A^−1^]^e^	PE_max_ [lm W^−1^]^f^	EQE_max/1000_ [%]^g^	CIE^h^
TCz‐XT	508	84	4.9	4052	71.1	37.6	24.9/20.8	0.22, 0.50
DMAC‐XT	520	82	4.5	9882	83.7	50.5	25.6/24.5	0.28, 0.58
DMAC‐XT‐TCz	516	83	4.5	7784	106.5	66.0	33.7/27.5	0.26, 0.56
HF device	526	35	3.9	4729	117.7	80.4	30.2/27.4	0.24, 0.69

^a^ peak value of electroluminescence.

^b^ full width at half maximum.

^c^ turn‐on voltage at 1 cd m^−2^.

^d^ maximum luminance.

^e^ maximum current efficiency.

^f^ maximum power efficiency.

^g^ maximum external quantum efficiency (EQE) and EQE value at 1000 cd m^−2^.

^h^ coordinates of Commission Internationale de L'Eclairage.

**FIGURE 6:**
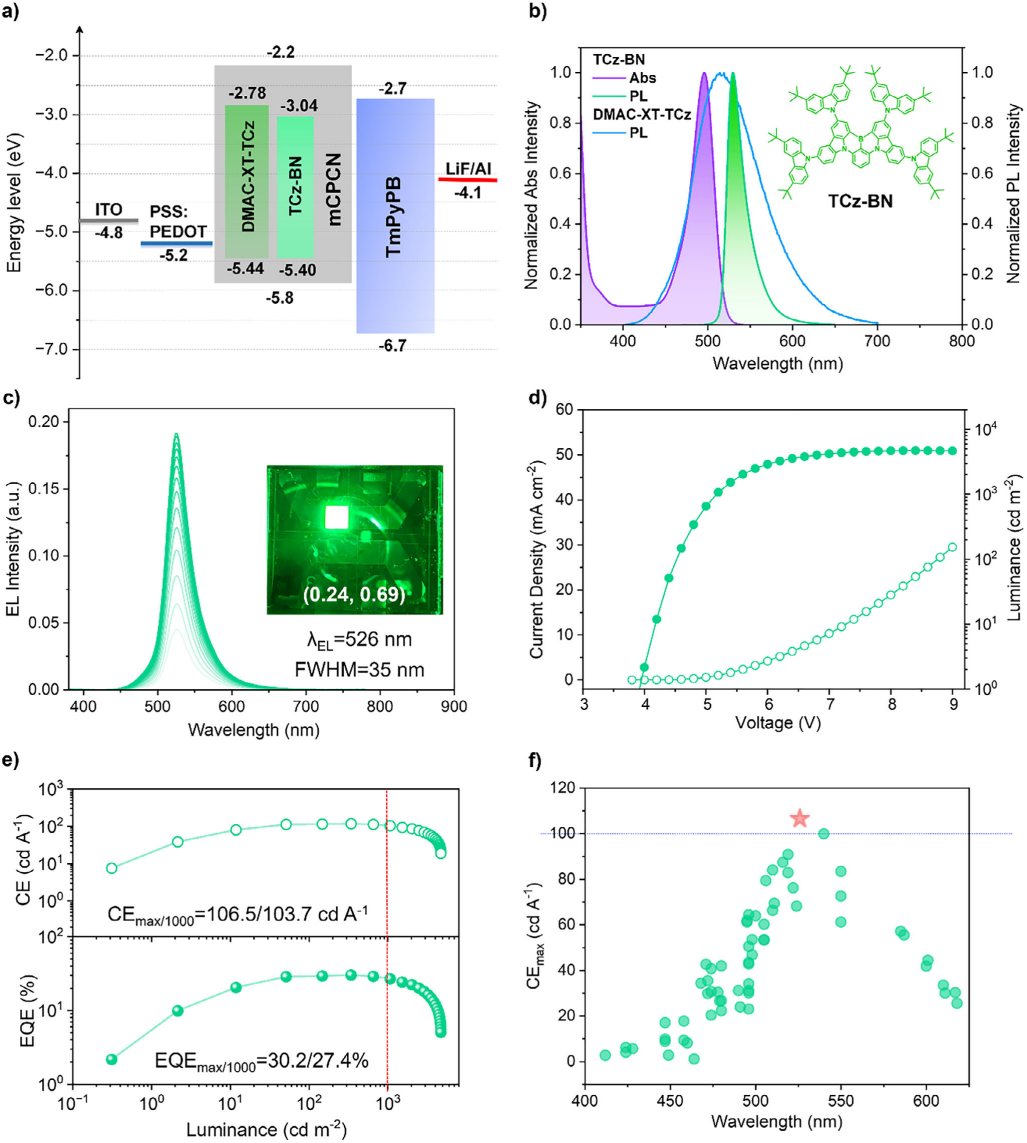
The EL performance of HF OLEDs employing **DMAC‐XT‐TCz** as sensitizer. (a) Energy diagram and the device architectures. (b) UV−vis absorption spectrum of **TCz‐BN** and the PL spectra of **TCz‐BN** and **DMAC‐XT‐TCz** in toluene solution. Inset: the molecular structure of **TCz‐BN**. (c) EL spectra of HF OLEDs. Inset: a photograph of HF OLEDs with CIE coordinate. (d) *J–V–L* curves. (e) The CE and EQE as a function of current density. (f) The CE_max_ summary of the representative solution‐processed OLEDs based on MR‐TADF emitters with emission peak from 400 to 625 nm.

Currently, solution‐processed hyperfluorescence (HF) OLEDs employing D‐A type TADF materials as sensitizers for multi‐resonance TADF (MR‐TADF) based OLEDs are considered as an encouraging technique, which potentially bring a new dawn for realizing high‐performance display with high color purities. Based on Förster energy transfer (FRET) from sensitizer to dopant with narrow full width at half maximum (FWHM), HF OLEDs can take full advantage of excellent exciton utilization of the sensitizers and the narrowband emission of the dopants [54, 55, 56]. Inspired by the excellent photophysical properties and device performances of the **DMAC‐XT‐TCz** based solution‐processed OLEDs, its applicability to serve as sensitizer in solution‐processed HF OLEDs was also evaluated. We first tested devices with **DMAC‐XT‐TCz**‐sensitized MR‐TADF molecules at different doping concentrations under the same device structure, and the optimal device configuration was ultimately determined to have 30 wt.% sensitizer and 2 wt.% MR‐TADF in emitting layer (Figure S29 and Table S2). Solution‐processed HF OLEDs with structure of ITO/PEDOT: PSS (30 nm)/EML (30 nm)/ TmPyPB (45 nm)/LiF (0.9 nm)/Al (110 nm) were fabricated as depicted in Figure 6a. In EML, the previously reported green MR‐TADF emitter **TCz‐BN** was adopted as dopant. Notably, the dominant absorption band of **TCz‐BN** is almost completely overlapped with the PL spectrum of **DMAC‐XT‐TCz** (Figure 6b), ensuring that the electrically generated energy of sensitizer can be efficiently transferred to the emissive state of MR‐TADF emitter with energy confinement. The spectrum stability was also evaluated as shown in Figure 6c. The profiles of EL spectra remain completely consistent without any extra emission from sensitizer or host materials in working voltage between 5–9 V. The maximum emission peak (*λ*_EL_ = 526 nm), FWHM (35 nm) and molecular structure of **TCz‐BN** are also provided here. According to the *J–V–L* curves (Figure 6d), the *V*_on_ and *L*_max_ of HF OLEDs are 3.9 V and 4729 cd m^−2^, respectively. Impressively, the HF devices achieved *EQE*_max_, *CE*_max_, and *PE*_max_ of 30.2%, 117.7 cd A^−1^ and 80.4 lm W^−1^ (Figure 6e; Figure S30). It should be noted that the 117.7 cd A^−1^ *CE*_max_ achieved by **DMAC‐XT‐TCz** sensitized HF devices represents the highest efficiency among the solution‐processed MR‐TADF OLEDs so far (Figure 6f; Table S4). The control device without sensitizer exhibited EQE_max_, CE_max_, and PE_max_ of 12.4%, 50.4 cd A^−1^ and 26.4 lm W^−1^, respectively, indicating that the *EQE*_max_ values in HF OLEDs are increased by more than twice than that in OLEDs without sensitizer. Especially at high luminance of 1000 cd m^−2^, the HF OLEDs can maintain superior EQE of 27.4%, which exceeds nearly sixfold compared with the control device. These results demonstrate the excellent performance of **DMAC‐XT‐TCz** as solution‐processed sensitizer for MR‐TADF emitter to boost exciton utilization especially in high current density.

## 3 Conclusion

In summary, a symmetry breaking strategy is adopted to construct an asymmetrical dendritic TADF emitter denoted as **DMAC‐XT‐TCz**. Another two symmetrical TADF molecules named **TCz‐XT** and **DMAC‐XT** as control are also synthesized. This strategy involves the deliberate excited‐state modulation. An in‐depth analysis on the photophysical properties combining with theoretical calculation expose that the asymmetrical architecture of asymmetric emitter **DMAC‐XT‐TCz** switches degenerated triplet excited states in **TCz‐XT** and **DMAC‐XT** to isolated counterpart, thereby effectively breaking the degeneracy of vibrational levels and boosting the RISC process. Additionally, the nonradiative decay is also suppressed to only 0.8 × 10^6^ s^−1^ due to imbedding oxygen linkage to locking electron‐donating skeleton, which is well consistent with the relatively lower RMSD value for **DMAC‐XT‐TCz**. As a result, near‐unity photoluminescence efficiency and excellent reverse intersystem crossing rate of 7.8 × 10^5^ s^−1^ can be achieved for **DMAC‐XT‐TCz**. Impressively, the optimized solution‐processed OLEDs achieve attractive EQE_max/1000_ of 33.7/27.5%, which are highest value for TADF dendrimer based OLEDs. By using **DMAC‐XT‐TCz** as sensitizer, the solution‐processed narrowband OLEDs based on a MR‐TADF emitter acquire dramatically improved device performances with *EQE*_max_ of 30.2% and record‐high current efficiency of 117.7 cd A^−1^. Our strategy accomplishes the boosted luminescence efficiency of TADF dendritic emitters through symmetry breaking, enabling the participation of multiple triplet states and enhanced SOC strength between singlet and triplet excited states, which achieves state‐of‐the‐art device performance both in solution‐processed OLEDs and in narrowband HF OLEDs.

## Funding

The National Natural Science Foundation of China (52103220, 52273164, and 62441403), the Shandong Provincial Natural Science Foundation (ZR2022ZD37 and ZR2023QE078), the Science and Technology Support Plan for Youth Innovation of Colleges and Universities in Shandong Province (2023KJ097), and the Natural Science Foundation of Qingdao (23‐2‐1‐75‐zyyd‐jch)

## Conflicts of Interest

The authors declare no conflicts of interest.

## Supporting information




**Supporting File**: advs73902‐sup‐0001‐SuppMat.docx.

## Data Availability

The data that support the findings of this study are available from the corresponding author upon reasonable request.
