# Supplementary material for: High‐Efficiency TADF Dendritic Emitters Enabled by Synchronously Inhibiting Degenerated Triplet Excited States and Structural Relaxation Toward Solution‐Processed OLEDs with EQE Over 33%
Source: Adv Sci (Weinh). 2026 Jan 18;13(18):e24183. doi: 10.1002/advs.202524183 (PMC13042566; doi:10.1002/advs.202524183)
Supplement: Supplementary file 1 — Supporting File: advs73902‐sup‐0001‐SuppMat.docx. [file ADVS-13-e24183-s001.docx]

Supporting Information

**High-Efficiency TADF Dendritic Emitters Enabled by Synchronously Inhibiting Degenerated Triplet Excited States and Structural Relaxation towards Solution-Processed OLEDs with EQE over 33%**

Xiaoxiang Yu, Wei Ping, Chengshuang Song, Hongxuan Liu, Qianyu Dong, Shuai Tang, Qilin Jiang, Lei Hua, Junjie Wang, Shian Ying, Yuchao Liu*, Zhongjie Ren*, Shouke Yan*

**Contents**

S1. Materials and experimental procedures

S2. Computational methods

S3. Device fabrication and measurement

S4. Analysis of rate constants

S5. Synthesis and characterization

S6. Thermal analysis

S7. Quantum chemical calculation

S8. Electrochemical analysis

S9. Photophysical analysis

S10. Morphology analysis of emitting films

S11. OLED performance

S12. Summary of reported device performances

S13. Optimized atomic coordinates

S14. Reference

**S1. Materials and experimental procedures**

All the reagents were purchased from *J&K Scientific*, *Energy Chemical, Sigma-Aldrich* and used without further purification, unless otherwise noted. The solvents were purchased from *Sinopharm Chemical Reagent Co., Ltd.*, and anhydrous toluene was gained through sodium reflux. The NMR spectra were performed using a Bruker AVAN CE NEO 400 spectrometer (400 MHz). ^1^HNMR and ^13^C NMR spectra were obtained with chloroform-*d* as solvent and TMS as internal standard, respectively. Electron spray mass spectra data (ESI-MS) were collected on Xevo G2 Qtof. Cyclic voltammetry (CV) was performed in nitrogen-bubbled acetonitrile solvent using CHI voltammetric analyser at room temperature. Tetrabutylammonium hexafluorophosphate (TBAPF_6_ 0.1 M) was adopted as the electrolyte, and a glassy carbon working electrode, a platinum wire auxiliary electrode, and an Ag/AgNO_3_ pseudo-reference electrode were used in the conventional three-electrode system. Cyclic voltammograms were performed from 0 V to 1.5 V at scan rate of 100 mV s^-1^, and ferrocene power as external standard^[1,2]^. Then the HOMO energy levels could be calculated according to the external reference ferroceneredox couple in acetonitrile by using the following formula^[3]^:

$$E_{\mathrm{HOMO}}=-\left( E_{\left( onset,ox vs {\mathrm{Fc}^{+}}/\mathrm{Fc} \right)}+4.8 \right)$$

The LUMO energy levels could be calculated by the HOMO values and the energy gap (*E*_g_s) obtained from the onset of the absorption spectra of the emitters.

Differential scanning calorimetry (DSC) was performed on a TA Q2000 differential scanning calorimeter at a heating rate of 10 °C min^-1^ from 30 to 300 °C under nitrogen atmosphere. The glass transition temperatures (*T*_g_) were determined from the second heating scan. Thermogravimetric analysis (TGA) was performed with a METTLER TOLEDO TGA/DSC 1/1100SF instrument. The thermal stability of the samples was obtained by measuring their 5% weight loss while heating at a rate of 10 °C min^-1^ from 25 to 800 °C under a nitrogen atmosphere.

UV-vis absorption spectra were recorded on a Hitachi U-2910 spectrophotometer. PL spectra, including fluorescence at 77 K, and phosphorescence at 77 K, were recorded on a Hitachi F-7000 fluorescence spectrophotometer, and the energy gap (Δ*E*_ST_) between lowest singlet (S_1_) and triplet excited states (T_1_) were determined from the difference values of the onset positions of fluorescent and phosphorescent spectra. To gain the proportion of delayed fluorescence (DF), the steady-state PL spectra in vacuum and in air, were performed using FS-5 spectrometer from Edinburgh Instruments Limited with Xe lamp source. The photoluminance quantum yields of the blended films were measured on FLS-980 with an integrating sphere (*φ* = 150 mm) under nitrogen gas. The transient-state PL spectra in vacuum and in air, including prompt fluorescence (PF) and DF spectra, and the temperature dependence of transient PL decay curves were determined using nanosecond gated luminescence and lifetime measurements with a high-energy pulsed Nd: YAG laser emitting at 320 nm.

**S2. Computational methods**

All the density functional theory (DFT) calculations were carried out using Gaussian 16 package. The following procedures were performed to optimize the molecular structures, calculate the energy properties and analyze the excited states of emitters. Firstly, the molecular structures were optimized in Gaussian 16 by performing a density functional theory (DFT) calculation in the PBE1PBE functional at the basis set level of 6-31G(d) for the ground state. Theoretical chemical calculations were performed by the Gaussian 16 program package under vacuum conditions without considering the influence of solvent effects. Then according to the optimized results, highest occupied molecular orbital (HOMO) and lowest unoccupied molecular orbital (LUMO) levels, and ground-state (S_0_) dipole moments can be obtained. The excited-state properties were obtained by time-dependent density functional theory (TD-DFT) at the basis set level of PBE1PBE/6-31G(d). In addition, the overlaps between the hole and electron density distributions in the S_1_ and T_1_ states were extracted by the Multiwfn code and visualized using VMD^[4, 5]^. To boost the calculation precision of the singlet and triplet energy levels, the number of calculated states was set to 10. Hence, the difference of dipole moments between S_0_ and S_1_, and transition dipole moments from S_1_ to S_0_ can be obtained from electron excitation analysis of TD-DFT calculations. The SOC matrix elements were assessed using PySOC. The Huang–Rhys factor and reorganization energy analysis were obtained by using the Molecular Materials Property Prediction Package. For the spin-orbit coupling calculations, the PBE0-D3/def2-TZVP level of theory was employed, whereas the reorganization energy calculations were carried out at the PBE1PBE/6-31G level.

Actually, in the TD-DFT calculations and simulations, we employed a variety of functions (including M062X, ωB97XD, PBE1PBE, and hybrid functions of DSDPBEP86 and B2PLYP) to perform the calculations of excited-state structure and energy levels on the basis of optimized results for **DMAC-XT-TCz**. As listed in **Table S1**, the data obtained from the M062X and PBE1PBE exhibit small energy splitting between S_1_ and T_1_ states (Δ*E*_ST_), 0.029 eV for M062X and 0.013 eV for PBE1PBE, respectively, which are well consistent with experimental results. In contrast, the calculated Δ*E*_ST_ values are 0.456 eV for ωB97XD, 1.0412 eV for DSDPBEP86 and 0.3427 eV for B2PLYP, all of which are overlarge for reverse intersystem crossing (RISC) process. To further conduct a systematic benchmarking of these functions, the higher-lying energy levels of triplet excited states and maximum emission peaks are also evaluated. For M062X and ωB97XD, the maximum emission peaks locate on 340 and 370 nm (**Figure Sx**). While the calculated maximum emission peak of **DMAC-XT-TCz** without any solvents is 491 nm by using PBE1PBE function, which is quite close the experimental result (515 nm in toluene). Therefore, we select the PBE1PBE function for excitation/emission energy calculations.

**Table S1**. The calculation functions of excited-state natures for **DMAC-XT-TCz**.

| Function | Excited-State Energy level  [eV] | | | Maximum peak  [nm] |
| --- | --- | --- | --- | --- |
|  | S_1_ | T_1_ | T_2_ |  |
| M062X | 3.362 | 3.333 | 3.349 | 340 |
| ωB97XD | 3.643 | 3.187 | 3.221 | 370 |
| PBE1PBE | 2.520 | 2.507 | 2.647 | 491 |
| DSDPBEP86 | 4.225 | 3.184 | 3.216 | - |
| B2PLYP | 3.628 | 3.285 | 3.319 | - |

**S3. Device fabrication and measurement**

Glass substrates pre-coated with a 100-nm-thin layer of indium tin oxide (ITO) with a sheet resistance of 20 Ω per square were thoroughly cleaned in ultrasonic bath of washing liquor, deionized water and isopropyl alcohol. Then the substrates were totally dried in a 120 °C oven. After that, the ITO substrates were treated by air plasma for 8 minutes. The hole transportation layer (HTL) was firstly performed by spin coating PEDOT: PSS 4083 on ITO substrates with 40 nm thickness. After that, the ITO substrates were transferred into glovebox, and heat treated for 30 min at 120 °C. Then 30-nm-thickness emitting layer was spin-coated on the HTL followed by heat treated for another 30 min at 60 °C. The ITO substrates were moved to vacuum evaporation chamber, and the residual functional layers were deposited sequentially by high-vacuum (<1.0 × 10^−5^ Pa) thermal evaporation onto a glass substrate pre-coated with hole transportation and emissive layers. The 30-nm-thickness electron transportation layer of TmPyPB, 0.9-nm-thickness electron injection layer of LiF and 110-nm-thickness cathode of Al layers were deposited with the rate of 2.0~2.5, 0.2 and 1~5 Å s^−1^, respectively. The active area of each device was 0.0625 cm^2^, and each device has four pixels. The electroluminescence spectra, the current density-voltage characteristics and the current density-voltage-luminance curves characterizations of the naked OLEDs were carried out with a Photo Research Spectra Scan PR-670 Spectroradiometer and a Keithley 2400 Source Meter and they are recorded simultaneously. All measurements were done at room temperature under ambient conditions.

According to the Marcus equation as following, $k_{RISC}$ is mainly related to three parameters: Δ*E*_ST_, spin-orbit coupling constant (SOCME) and recombination energy (λ). These three parameters of the molecule can be obtained through quantum chemical calculations, and then the RISC process can be quantified *k*_RISC_ values, so as to reflect the optoelectronic performance of the molecules.

$$\begin{aligned} k_{RISC}=\frac{2\pi}{\hbar}\nu^{2}\sqrt{\frac{1}{4\pi k_{B}T\lambda}}exp\left[ -\frac{\left( {\Delta E}_{ST}+\lambda\right)^{2}}{4k_{B}T\lambda} \right] \end{aligned}$$

$\hbar$ represents the reduced Planck constant; ν represents the SOCME value; *k*_B_ is the Boltzmann constant; T is the temperature (300 K). Therefore, the *k*_RISC_ values can be calculated to be 2.34×10^3^ s^-1^ (S_1_-T_1,2_) for **TCz-XT**, 5.64×10^5^ s^-1^ (S_1_-T_1,2_) for **DMAC-XT**, 5.67×10^4^ s^-1^ (S_1_-T_1,2_) for **DMAC-XT-TCz**. Considering the fact that the emission core of **DMAC-XT-TCz** is located on DMAC-XT segment, the λ value of **DMAC-XT** is approximatively adopted, and thus the reformative *k*_RISC_ value of **DMAC-XT-TCz** is 1.04×10^5^ s^-1^ (S_1_-T_1,2_).

**S4. Analysis of rate constants**

Time-resolved transient photoluminescence decay measurements were utilized to study the delayed fluorescence phenomenon of the molecule in doped films (30 wt% in DPEPO), Rate constants of different kinetic processes were calculated following the equations (S1)-(S3) below: where $k_{r}^{S}$, $k_{nr}^{S}$, $k_{\mathrm{RISC}}$ represent the rate constant of singlet radiative decay, non-radiative decay of singlet radiative decay, and reverse intersystem crossing, respectively; $\emptyset_{\mathrm{PL}}$, $\varphi_{\mathrm{DF}}$, *τ*_p_ and *τ*_d_ represent total PLQY, quantum yield of the delayed component, lifetimes of the prompt and delayed components, respectively.

In this study, $\varphi_{\mathrm{DF}}$ and the quantum yield of the delayed component ($\varphi_{\mathrm{PF}}$) were determined by using the total PLQY and the integrated intensity ratio between prompt and delayed components which was calculated from transient photoluminescence measurements as equations (S4)-(S6) below:

| ${{k_{\mathrm{RISC}}=\emptyset}_{\mathrm{PL}}}/\left[ \tau_{d}\left( 1-\varphi_{\mathrm{DF}} \right) \right]$ | S1 |
| --- | --- |
| ${{k_{r}^{S}=(1-\varphi}_{\mathrm{DF}})}/{\tau_{p}}$ | S2 |
| $k_{nr}^{S}=k_{r}^{S}\times\left( 1-\emptyset_{\mathrm{PL}} \right)/{\emptyset_{\mathrm{PL}}}$ | S3 |
| $\text{I (t)=}\text{A}_{\text{p}}\text{e}^{\text{-}\frac{\text{t}}{\text{τ}_{\text{p}}}}$ + $\text{A}_{\text{d}}\text{e}^{\text{-}\frac{\text{t}}{\text{τ}_{\text{d}}}}$ | S4 |
| $\varphi_{\mathrm{PF}}\text{=}\frac{\text{A}_{\text{p}}\text{τ}_{\text{p}}}{\text{A}_{\text{p}}\text{τ}_{\text{p}}\text{+}\text{A}_{\text{d}}\text{τ}_{\text{d}}}$ | S5 |
| $\varphi_{\mathrm{DF}}\text{=}\frac{\text{A}_{\text{d}}\text{τ}_{\text{d}}}{\text{A}_{\text{p}}\text{τ}_{\text{p}}\text{+}\text{A}_{\text{d}}\text{τ}_{\text{d}}}$ | S6 |

**S5. Synthesis and characterization**

**Scheme S1.** Synthesis route of methyl 2-(3-iodophenoxy)-4-bromobenzoate.

3-Iodophenol (1.1 g, 5 mmol) and methyl 4-bromo-2-fluorobenzoate (1.28 g, 5.5 mmol) were added into two-necked flask under nitrogen atmosphere, and then anhydrous DMF (60 mL) was injected into mixture. After stirring at 90 °C for 12 h, the reaction was cooled down to room temperature. Then the mixture was extracted with dichloromethane/water for three times, and then organic layer was dehydrated with anhydrous magnesium sulfate. After filtering and drying, crude product could be obtained. The crude product was purified by column chromatography (the eluent was dichloromethane: petroleum ether = 1:5). Target compound of methyl 2-(3-iodophenoxy)-4-bromobenzoate was obtained as a colorless oily liquid in 82% yield (1.77 g).

^1^H NMR (400M Hz, CDCl_3_) δ 7.71 (d, *J* = 8.4 Hz, 1H), 7.36 (dt, *J* = 7.8, 1.2 Hz, 1H), 7.29-7.21 (m, 2H), 7.04 (d, *J*=1.9Hz, 1H), 6.96 (t, *J* = 8.0 Hz, 1H), 6.84 (dd, *J* = 8.2, 2.3 Hz, 1H), 3.72 (s, 3H).

^13^C NMR (101 MHz, CDCl_3_) δ 165.06, 157.47, 156.14, 133.24, 132.83, 131.23, 127.64, 127.41, 127.35, 124.14, 121.98, 117.64, 94.49, 52.43.

**Scheme S2.** Synthesis route of methyl 2-(3-iodophenoxy)-4-bromobenzoicacid.

Methyl 2-(3-iodophenoxy)-4-bromobenzoate (2.77 g, 6.37 mmol) was dissolved in ethanol (40 mL) and potassium hydroxide (3.57 g, 63.7 mmol) aqueous solution (20 mL) were added into two-necked flask under nitrogen atmosphere. After stirring at 80℃ for 12 h, the reaction was cooled down to room temperature. Then the mixture was extracted with dichloromethane/water for three times, and then organic layer was dehydrated with anhydrous magnesium sulfate. After filtering and drying, crude product could be obtained. The crude product was purified by column chromatography (the eluent was dichloromethane: petroleum ether=1:1). Methyl 2-(3-iodophenoxy)-4-bromobenzoicacid was obtained as a brown solid in 98% yield (2.44 g).

^1^H NMR (400M Hz, DMSO-*d*_6_) δ 13.13 (s, 1H), 7.79 (t, *J* = 8.8 Hz, 1H), 7.54 (dd, *J* = 8.4, 1.9 Hz, 1H), 7.50-7.45 (m, 1H), 7.34-7.27 (m, 2H), 7.16 (t, *J* = 8.0 Hz, 1H), 7.01-6.89 (m, 1H).

^13^C NMR (101 MHz, DMSO) δ 168.64, 159.58, 153.35, 132.56, 131.79, 131.19, 130.12, 127.67, 126.15, 124.41, 120.86, 117.22, 95.15.

**Scheme S3.** Synthesis route of 3-bromo-6-iodo-9H-xanthen-9-one (Br-I-XT).

Methyl 2-(3-iodophenoxy)-4-bromobenzoicacid (1.18 g, 2.7 mmol) dissolved into concentrated sulfuric acid (30 mL) were added into two-necked flask. After stirring at 110℃ for 1 h, the reaction was cooled down to room temperature and then the reaction mixture was poured into an ice slurry. Sodium hydroxide was used to adjust the PH value to 9 and then plenty of white solid was precipitated. Then the mixture was vacuum filtration and the solid was extracted with dichloromethane/water for three times, and then organic layer was dehydrated with anhydrous magnesium sulfate. After filtering and drying, crude product could be obtained. The crude product was purified by column chromatography (the eluent was dichloromethane: petroleum ether=1:10), Compound of Br-I-XT was obtained as a pale yellow solid in 75% yield (800 mg).

^1^H NMR (400M Hz, CDCl_3_) δ 8.18 (d, *J* = 8.5 Hz, 1H), 8.00 (d, *J* = 8.4 Hz, 1H), 7.92 (d, *J* = 1.5Hz, 1H), 7.74 (dd, *J* = 8.4, 1.6 Hz, 1H), 7.69 (d, *J* = 1.8 Hz, 1H), 7.53 (dd, *J* = 8.5, 1.8 Hz, 1H).

^13^C NMR (101 MHz, CDCl_3_) δ 176.02, 155.86, 155.58, 133.78, 129.60, 128.12, 128.05, 127.83, 127.19, 121.20, 121.15, 120.72, 101.95.

TOF-MS C13H6BrIO2 Calculated [M+H]: 400.8674; Obtained [M+H]: 400.8674.

**Scheme S4.** Synthesis route of 3,6-bis[3,3'',6,6''-tetra-tert-butyl-9'H-9,3':6',9''-tercarbazole]-9H-xanthen-9-one (**TCz-XT**).

Compound of Br-I-XT (995 mg, 1 mmol), 3,3'',6,6''-tetra-tert-butyl-9'H-9,3':6',9''-tercarbazole (TCz) (1588.4 mg, 2.2 mmol), Pd_2_(dba)_3_ (91.7 mg, 0.10 mmol), [(*t*-Bu)_3_PH]BF_4_ (58 mg, 0.2 mmol) and *t*BuONa (240.3 mg, 2.5 mmol) were added into two-necked flask under nitrogen atmosphere, and then anhydrous toluene (60 mL) was injected into mixture. After stirring at 110℃ for 12 h, the reaction was cooled down to room temperature. Then the mixture was extracted with dichloromethane/water for three times, and then organic layer was dehydrated with anhydrous magnesium sulfate. After filtering and drying, crude product could be obtained. The crude product was purified by column chromatography (the eluent was dichloromethane: petroleum ether=1:3). **TCz-XT** was obtained as a yellow-green solid in 82% yield (1341.7 mg).

^1^H NMR (400 MHz, CDCl_3_) δ 8.71 (d, J = 8.5 Hz, 1H), 8.58 (d, J = 8.5 Hz, 1H), 8.26 (d, J = 2.0 Hz, 2H), 8.16 (d, J = 1.9 Hz, 4H), 7.92 (d, J = 1.9 Hz, 1H), 7.84 (dd, J = 11.2, 8.7 Hz, 3H), 7.67 (dd, J = 8.7, 2.0 Hz, 2H), 7.59 (d, J = 2.0 Hz, 1H), 7.52 (dd, J = 7.5, 1.7 Hz, 2H), 7.46 (dd, J = 8.6, 2.0 Hz, 5H), 7.34 (d, J = 8.6 Hz, 4H), 7.12 – 7.03 (m, 4H), 6.68 (dd, J = 7.9, 1.5 Hz, 2H), 1.68 (s, 6H), 1.46 (s, 36H).

^13^C NMR (101 MHz, CDCl_3_) δ 142.75, 140.30, 140.01, 139.48, 133.81, 131.91, 129.40, 129.14, 126.50, 126.34, 125.32, 124.79, 123.83, 123.66, 123.23, 122.52, 122.40, 119.48, 116.89, 116.30, 115.87, 115.32, 111.28, 109.05, 34.77, 32.06, 31.47, 30.22, 29.72.

TOF-MS: C117H114N6O2 Calculated [M+H]: 1634.9003; Obtained [M+H]: 1634.9003.

**Scheme S5.** Synthesis route of 3,6-bis[9,9-dimethyl-10(9H)-acridinyl]-9H-xanthen-9-one (**DMAC-XT**).

Compound of Br-I-XT (995 mg, 1 mmol), 9,9-dimethyl-9,10-dihydroacridine (DMAC) (460.2 mg, 2.2 mmol), Pd_2_(dba)_3_ (91.7 mg, 0.10 mmol), [(*t*-Bu)_3_PH]BF_4_ (58 mg, 0.2 mmol) and *t*BuONa (240.3 mg, 2.5 mmol) were added into two-necked flask under nitrogen atmosphere, and then anhydrous toluene (60 mL) was injected into mixture. After stirring at 110℃ for 12 h, the reaction was cooled down to room temperature. Then the mixture was extracted with dichloromethane/water for three times, and then organic layer was dehydrated with anhydrous magnesium sulfate. After filtering and drying, crude product could be obtained. The crude product was purified by column chromatography (the eluent was dichloromethane: petroleum ether=1:3). **DMAC-XT** was obtained as a yellow-green solid in 90% yield (550.0 mg).

^1^H NMR (400 MHz, CDCl_3_) δ 8.55 (d, J = 8.4 Hz, 2H), 7.60 – 7.45 (m, 6H), 7.41 (dd, J = 8.5, 1.9 Hz, 2H), 7.04 (pd, J = 7.3, 1.7 Hz, 8H), 6.56 (dd, J = 7.6, 1.8 Hz, 4H), 1.68 (s, 12H).

^13^C NMR (101 MHz, CDCl_3_) δ 175.72, 157.92, 148.33, 140.27, 132.97, 129.38, 126.48, 125.31, 124.41, 122.16, 120.43, 116.96, 116.22, 36.48, 30.45.

TOF-MS: C43H34N2O2 Calculated [M+H]: 610.2620; Obtained [M+H]: 610.2625.

**Scheme S6.** Synthesis route of 3-bromo-6-bis[3,3'',6,6''-tetra-tert-butyl-9'H-9,3':6',9''-tercarbazole]-9H-xanthen-9-one (**Br-XT-TCz**).

Compound of Br-I-XT (401 mg, 1 mmol), TCz (230 mg, 1.1 mmol), Pd(OAc)_2_ (13.5 mg, 0.06 mmol), P(*t*-Bu)_3_ (20.2 mg, 0.1 mmol) and *t*BuONa (240.3 mg, 2.5 mmol) were added into two-necked flask under nitrogen atmosphere, and then anhydrous toluene (60 mL) was injected into mixture. After stirring at 80℃ for 12 h, the reaction was cooled down to room temperature. Then the mixture was extracted with dichloromethane/water for three times, and then organic layer was dehydrated with anhydrous magnesium sulfate. After filtering and drying, crude product could be obtained. The crude product was purified by column chromatography (the eluent was dichloromethane: petroleum ether=1:3). Compound of **Br-XT-TCz** was obtained as a yellow-green solid in 85% yield (844 mg).

^1^H NMR (400M Hz, CDCl_3_) δ 8.58 (d, *J* = 8.5 Hz, 1H), 8.23-8.17 (m, 3H), 8.09 (d, *J* = 1.9 Hz, 4H), 7.85 (d, *J* = 1.9 Hz, 1H), 7.80-7.69 (m, 4H), 7.60 (dd, *J* = 8.7, 2.0 Hz, 2H), 7.51 (dd, *J* = 8.5, 1.8 Hz, 1H), 7.39 (dd, *J* = 8.7, 1.9 Hz, 4H), 7.27 (d, *J* = 8.6 Hz, 4H), 1.39 (s, 36H).

^13^C NMR (101 MHz, CDCl_3_) δ 142.77, 139.99, 139.42, 131.98, 129.14, 128.29, 128.23, 126.35, 124.83, 123.66, 123.24, 122.59, 121.21, 119.49, 116.31, 115.34, 111.24, 109.03, 34.76, 32.05, 29.73.

TOF-MS: C65H60BrN3O2 Calculated [M+H]: 994.3947; Obtained [M+H]: 994.3942.

**Scheme S7.** Synthesis route of 3-9,9-dimethyl-10(9H)-acridinyl-6-3,3'',6,6''-tetra-tert-butyl-9'H-9,3':6',9''-tercarbazole-9H-xanthen-9-one (**DMAC-XT-TCz**).

Compound of **Br-XT-TCz** (995 mg, 1 mmol), DMAC (251 mg, 1.2 mmol), Pd_2_(dba)_3_ (55 mg, 0.06 mmol), [(*t*-Bu)_3_PH]BF_4_ (29 mg, 0.1 mmol) and *t*BuONa (240.3 mg, 2.5 mmol) were added into two-necked flask under nitrogen atmosphere, and then anhydrous toluene (60 mL) was injected into mixture. After stirring at 110℃ for 12 h, the reaction was cooled down to room temperature. Then the mixture was extracted with dichloromethane/water for three times, and then organic layer was dehydrated with anhydrous magnesium sulfate. After filtering and drying, crude product could be obtained. The crude product was purified by column chromatography (the eluent was dichloromethane: petroleum ether=1:3). **3CZ-XT-DMAC** was obtained as a yellow-green solid in 85% yield (954 mg).

^1^H NMR (400 MHz, CDCl3) δ 8.71 (d, J = 8.5 Hz, 1H), 8.58 (d, J = 8.5 Hz, 1H), 8.26 (d, J = 2.0 Hz, 2H), 8.16 (d, J = 1.9 Hz, 4H), 7.92 (d, J = 1.9 Hz, 1H), 7.84 (dd, J = 11.2, 8.7 Hz, 3H), 7.67 (dd, J = 8.7, 2.0 Hz, 2H), 7.59 (d, J = 2.0 Hz, 1H), 7.52 (dd, J = 7.5, 1.7 Hz, 2H), 7.46 (dd, J = 8.6, 2.0 Hz, 5H), 7.34 (d, J = 8.6 Hz, 4H), 7.12 – 7.03 (m, 4H), 6.68 (dd, J = 7.9, 1.5 Hz, 2H), 1.68 (s, 6H), 1.46 (s, 36H).

^13^C NMR (101 MHz, CDCl3) δ 173.03, 158.01, 157.35, 148.81, 143.27, 142.75, 140.30, 140.01, 139.48, 133.82, 131.91, 129.40, 129.14, 129.00, 128.43, 126.50, 126.34, 125.32, 124.79, 123.83, 123.66, 123.23, 122.52, 122.40, 119.48, 116.89, 116.30, 115.87, 115.32, 111.28, 109.05, 34.77, 32.06, 30.22, 29.72.

TOF-MS: C80H74N4O2 Calculated [M+H]: 1122.5812; Obtained [M+H]: 1122.5819.

**Figure S1**. ^1^H NMR spectrum (400 MHz) of methyl 2-(3-iodophenoxy)-4-bromobenzoate in CDCl_3_.

**Figure S2**. ^13^C NMR spectrum (400 MHz) of methyl 2-(3-iodophenoxy)-4-bromobenzoate in CDCl_3_.

**Figure S3**. ^1^H NMR spectrum (400 MHz) of methyl 2-(3-iodophenoxy)-4-bromobenzoicacid in CDCl_3_.


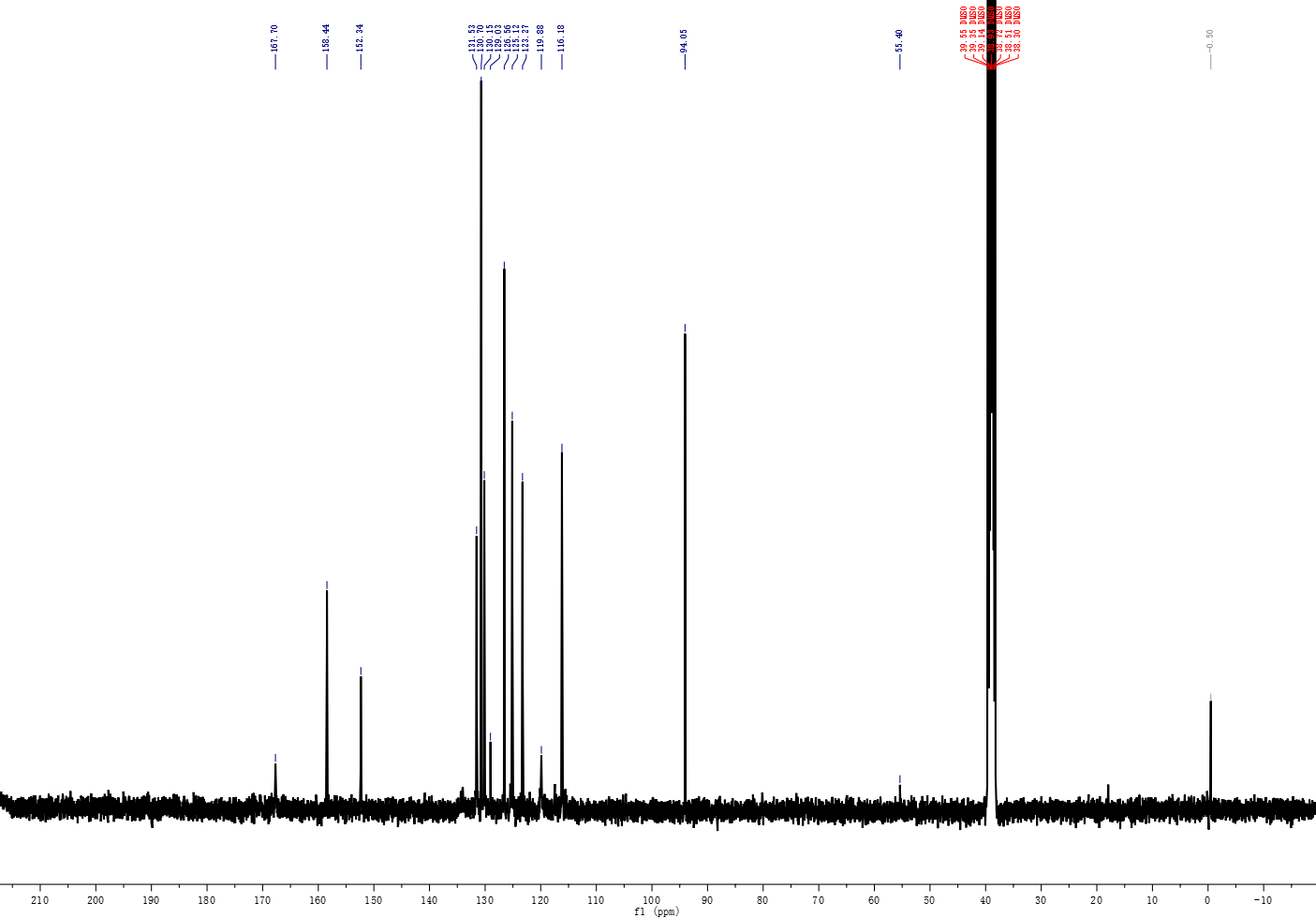


**Figure S4**. ^13^C NMR spectrum (400 MHz) of methyl 2-(3-iodophenoxy)-4-bromobenzoicacid in CDCl_3_.

**Figure S5**. ^1^H NMR spectrum (400 MHz) of Br-I-XT in CDCl_3_.

**Figure S6**. ^13^C NMR spectrum (400 MHz) of Br-I-XT in CDCl_3_.

**Figure S7**. TOF MS spectrum of Br-I-XT.

**Figure S8**. ^1^H NMR spectrum (400 MHz) of **TCz-XT** in CDCl_3_.

**Figure S9**. ^13^C NMR spectrum (400 MHz) of **TCz-XT** in CDCl_3_.

**Figure S10**. TOF MS spectrum of **TCz-XT**.

**Figure S11**. ^1^H NMR spectrum (400 MHz) of **DMAC-XT** in CDCl_3_.

**Figure S12**. ^13^C NMR spectrum (400 MHz) of **DMAC-XT** in CDCl_3_.


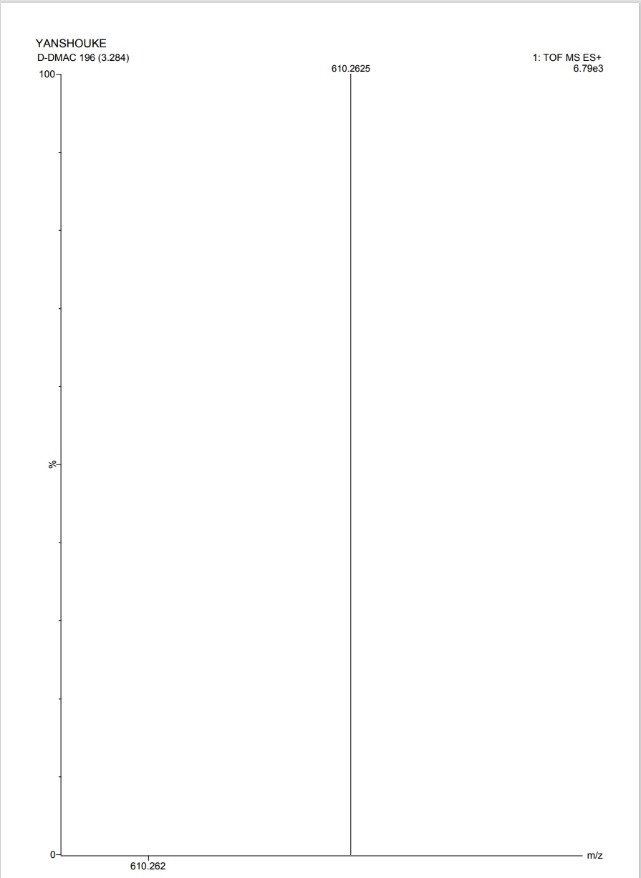


**Figure S13**. TOF MS spectrum of **DMAC-XT**.

**Figure S14**. ^1^H NMR spectrum (400 MHz) of **Br-XT-TCz** in CDCl_3_.

**Figure S15**. ^13^C NMR spectrum (400 MHz) of **Br-XT-TCz** in CDCl_3_.

**Figure S16**. TOF MS spectrum of **Br-XT-TCz**.

**Figure S17**. ^1^H NMR spectrum (400 MHz) of **DMAC-XT-TCz** in CDCl_3_.

**Figure S18**. ^13^C NMR spectrum (400 MHz) of **DMAC-XT-TCz** in CDCl_3_.

**Figure S19**. TOF-MS spectrum of **DMAC-XT-TCz**.

**S6. Thermal analysis**

**Figure S20.** The decomposition temperatures of (a) **TCz-XT**, (b) **DMAC-XT**, and (c) **DMAC-XT-TCz** obtained from TGA analyses.

**S7. Quantum chemical calculation**

**Figure S21**. (a) Molecular structures of previous works and structures of the newly-designed **TCz-XT**, **DMAC-XT**, and **DMAC-XT-TCz** reported in this study. The SOC matrix elements and RMSD values are also provided.

**Figure S22.** Root mean squared displacement (RMSD) between optimized S_0_ (yellow) and S_1_ (blue) geometries of (a) **tBuG2B**, (b) **DMAC-BP**, (c) **DMAC-BP-tBu3Cz**, (d) **TCz-XT**, (e) **DMAC-XT**, and (f) **DMAC-XT-TCz.**

**Figure S23.** Energy levels of fragements containg XT, DMAC, TCz.

**Figure S24**. The calculated energy leveles for **TCz-XT**, **DMAC-XT**, and **DMAC-XT-TCz**.

**Figure S25**. The calculated PL spectra of **DMAC-XT-TCz** by using different functions without any solvents.

**S8. Electrochemical analysis**

**Figure S26.** The oxidation potential of (a) **TCz-XT**, (b) **DMAC-XT**, and (c) **DMAC-XT-TCz** emitters determined by cyclic voltammetry measurement. (d) The *E*_1/2_ value of Fc^+^/Fc external standard.

**S9. Photophysical analysis**

**Figure S27.** The Lippert-Mataga plot (ν_abs_-ν_em_ against orientation polarizability of solvent) of (a) **TCz-XT**, (b) **DMAC-XT**, and (c) **DMAC-XT-TCz**, respectively.

**S10. Morphology analysis of emitting films**

**Figure S28**. AFM height images of neat films for (a) **TCz-TX**, (b) **DMAC-TX**, and (c) for **DMAC-XT-TCz**. The RMS values are also presented in diagram.

**S11. OLED performance**

**Figure S29.** The EL performance of solution-processed OLEDs employing DMAC-XT-TCz with doping ratios of 5, 15, 20, 25 and 30 wt%. (a) Energy diagram and the device architectures. (b) EL spectra of solution-processed OLEDs. (c) *J–V–L* curves. (d) The CE_max_ and PE_max_ as a function of current density. (e) The EQE_max_ as a function of luminance. (f) The EQE_max_ values at doping ratios of 5, 10, 15, 20, 25 and 30 wt%.

**Figure S30.** The EL performance of solution-processed OLEDs employing **DMAC-XT-TCz** as sensitizer with different doping ratios. (a) EL spectra of solution-processed OLEDs. (b) *J–V–L* curves. (c) The CE_max_ and PE_max_ as a function of current density. (d) The EQE_max_ as a function of luminance.

**Figure S31.** The PE of **DMAC-XT-TCz** based hyperfluorescence OLED as a function of current density.

**Table S2.** The electroluminescence properties of sensitized OLEDs.

| Emitter | λ_EL_  [nm]*^a^* | FWHM  [nm]*^b^* | V_on_  [V]*^c^* | L_max_  [cd m^−2^]*^d^* | CE_max_  [cd A^-1^]*^e^* | PE_max_  [lm W^-1^]*^f^* | EQE_max_  [%]*^g^* | CIE*^h^* |
| --- | --- | --- | --- | --- | --- | --- | --- | --- |
| **10%DMAN-XT-TCz in 1%TCzBN** | 524 | 34 | 4.8 | 4660 | 88.1 | 48.3 | 23.2 | 0.22, 0.68 |
| **10%DMAN-XT-TCz in 2%TCzBN** | 528 | 34 | 4.4 | 5271 | 117.3 | 76.1 | 29.4 | 0.24, 0.70 |
| **15%DMAN-XT-TCz in 1%TCzBN** | 524 | 36 | 4.4 | 7171 | 87.3 | 54.9 | 23.2 | 0.24, 0.67 |
| **15%DMAN-XT-TCz in 2%TCzBN** | 526 | 35 | 4.4 | 7376 | 87.6 | 54.2 | 22.4 | 0.24, 0.69 |
| **20%DMAN-XT-TCz in 1%TCzBN** | 524 | 38 | 4.2 | 7998 | 85.3 | 55.8 | 23.3 | 0.24, 0.67 |
| **20%DMAN-XT-TCz in 2%TCzBN** | 528 | 35 | 4.4 | 5438 | 118.7 | 77.7 | 29.9 | 0.24, 0.70 |
| **30%DMAN-XT-TCz in 2%TCzBN** | 526 | 35 | 4.0 | 4729 | 117.7 | 78.4 | 30.2 | 0.24, 0.70 |

*^a^* peak value of electroluminescence;

*^b^* full width at half maximum;

*^c^* turn-on voltage at 1 cd m^−2^;

*^d^* maximum luminance;

*^e^* maximum current efficiency;

*^f^* maximum power efficiency;

*^g^* maximum external quantum efficiency (EQE)，

*^h^* coordinates of Commission Internationale de L’Eclairage.

**S12. Summary of reported device performances**

**Table S3**. The summary of EL properties for reported TADF dendrimer based solution-porcessed OLEDs.

| Emitter | V^[a]^  (V) | L_Max_  (cd m^-2^) | η_CE_  (cd A^-1^) | η_PE_  (lm W^-1^) | η_EQE/at1000_  (%) | λ (nm)/  CIE(x,y) | Ref |
| --- | --- | --- | --- | --- | --- | --- | --- |
| BSeN-DCz | ~5.0 | - | 27.0 | 19.8 | 19.1 | 479/  (0.11,0.24) | 6 |
| BSeN-TCz | ~4.5 | - | 26.9 | 21.9 | 19.7 | 478/  (0.11,0.21) | 6 |
| Cz-3CzBN:Cz-4CzIPN | 5.8 | 23000 | 73.4 | 46.1 | 24.3 | 551/  (0.32,0.56) | 7 |
| DCz-DPS-Cz | 3.1 | - | 62.0 | 51.6 | 23.3 | 498/  (0.23,0.42) | 8 |
| DCz-DPS-TCz | 3.0 | - | 65.9 | 59.2 | 24.0 | 500/  (0.24,0.45) | 8 |
| TBuG2TAZ | 2.5 | 2003 | 43.5 | 42.7 | 16.1 | 502/  (0.28,0.49) | 9 |
| tBuCz2pTRZ | 3.1 | ~3000 | 49.4 | 45.7 | 18.5 | 500/  (0.23, 0.46) | 10 |
| tBuCz2mTRZ | 3.3 | 777 | 59.9 | 52.2 | 19.9 | 516/  (0.27, 0.53) | 10 |
| tBuCz2m2pTRZ | 3.1 | 6029 | 98.8 | 91.3 | 28.7 | 540/  (0.37, 0.57) | 10 |
| 5CzBN-ESF | 3.4 | 17230 | 90.8 | 71.3 | 30.6/20.5 | 508/  (0.27, 0.55) | 11 |
| 5CzBN-BSF | 3.5 | 12366 | 77.6 | 58.0 | 26.5 | 508/  (0.26, 0.54) | 11 |
| 5CzBN-HSF | 3.5 | 12392 | 59.9 | 48.3 | 20.9 | 508/  (0.25, 0.54) | 11 |
| 5CzBN-Hex | 2.9 | 7464 | 38.6 | 35.7 | 13.1 | 508/  (0.28, 0.54) | 11 |
| 2CzphQAO | 3.8 | 1586 | 17.1 | - | 12.1 | 450/  (0.13, 0.14) | 12 |
| 43Cz-SO | 5.1 | 8135 | 36.2 | - | 12.6 | 447/  (0.24, 0.45) | 13 |
| 5CzTRZ-Cz | 3.5 | 500 | 70.6 | 49.3 | 23.3/21.5 | 534/  (0.31, 0.53) | 14 |
| DMAC-BP-tBuCz | 3.4 | 8024 | 64.7 | 43.4 | 20.9/20.4 | 520/  (0.28, 0.52) | 15 |
| DMAC-BP-tBu3Cz | 3.6 | 8543 | 87.8 | 68.9 | 28.0/24.9 | 520/  (0.28, 0.52) | 15 |
| DMAC-BP-tBu7Cz | 3.6 | 4619 | 84.3 | 66.2 | 26.5/17.6 | 520/  (0.29, 0.52) | 15 |
| 2FBP2GCz | 3.0 | - | 68.2 | 51.0 | 18.7/17.3 | 540/  (0.34, 0.60) | 16 |
| 2DPACzBP | 3.4 | - | 46.1 | 36.7 | 20.0/11.8 | 605/  (0.53, 0.45) | 16 |
| 2FBP2DPACz | 3.2 | - | 49.5 | 27.3 | 17.0/16.8 | 580/  ( 0.49, 0.49) | 16 |
| C-D1 | 3.2 | 1441 | 24.9 | - | 10.1/2.8 | 412/  (0.21, 0.35) | 17 |
| O-D1 | 3.0 | 3525 | 50.0 | - | 16.8/7.8 | 515/  (0.26, 0.50) | 17 |
| O-D2 | 3.0 | 8963 | 63.3 | - | 20.6/18.2 | 541/  (0.30, 0.52) | 17 |
| G-OmCP | 3.83 | 7739 | 56.34 | 44.25 | 19.9/  4.79 | 556 | 18 |

**Table S4**. The summary of EL properties for reported MR-TADF based solution-porcessed OLEDs.

| Emitter | λ_EL_  [nm] | FWHM  [nm] | EQE_max/100/1000_  [%] | CE_max_  (cd A^-1^) | Ref |
| --- | --- | --- | --- | --- | --- |
| OAB-ABP-1 /Polymer B | 505 | 33 | 21.8/19.6/17.4 | 53.3 | [19] |
| TB-3Cz  nondoped | 424 | 33.8 | 9.9/-/- | 4.0 | [20] |
| V-DABNA-Mes  /Polymer C | 480 | 27 | 22.9/20.3/10.9 | 26.7 | [21] |
| TB-tCz  /mCP | 412 | 44.2 | 15.9/-/- | 2.72 | [22] |
| TB-tPCz/ Nondoped | 428 | 42.2 | 15.8/-/- | 5.61 |  |
| BICz  Nondoped | 424 | 61 | 10.1/-/- | 6.13 | [23] |
| CzBN/CzAcSF | 480 | 35 | 14.7/-/- | 22.3 | [24] |
| BCzBN/CzAcSF | 490 | 32 | 16.3/-/- | 31.1 |  |
| *m*-Cz-BNCz/D2-OBu | 519 | 42 | 24.2/23.2/17.7 | 90.8 | [25] |
| TBN-TPA/mCP | 464 | 32 | 1.08/-/- | 1.08 | [26] |
| ν-DABNA/Au-1/PYD-2 | 480 | 23 | 16.6/-/14.4 | - | [27] |
| 6TBN/mCP | 496 | 25 | 23.0/16.5/6.4 | 42.8 | [28] |
| SpDCz/mCP | 498 | 32 | 18.6/13.3/- | 46.8 | [29] |
| DSpDCz/mCP | 505 | 31 | 19.0/13.8/- | 53.4 |  |
| BN-R  Without sensitizer | 617 | 47 | 22.0 | 30.2 | [30] |
| BN-R  With sensitizer | 618 | 47 | 20.1/16.4/13.3 | 25.5 |  |
| BNB′-1/PhCzBCz/4CzIPN | 540 | 30 | 36.2/-/- | 99.9 | [31] |
| BN-27Cz-BN  /DMIC-TRZ | 498 | 33 | 20.9/-/3.3 | 53.5 | [32] |
| BN-36Cz-BN  /DMIC-TRZ | 495 | 31 | 27.1/-/2.9 | 61.8 |  |
| Cz-DABNA  /5CzTRZ/mCP | 472 | 16.6 | 23.8/-/21.2 | 29.8 | [33] |
| tBuCz-DABNA  /5CzTRZ/mCP | 472 | 16.6 | 29.2/-/26.0 | 35.4 |  |
| TriBNCz/5TCzBN  /mCP | 496 | 30 | 28.8/-/26.1 | 64.4 | [34] |
| TCzBN-S/ SF3TRZ | 500 | 39 | 23.3/20.4/6.7 | 63.9 | [35] |
| TCzBN-SO/SF3TRZ | 516 | 41 | 25.5/19.3/6.9 | 87.4 |  |
| 3CzSF-BN/PhCzBCz | 522 | 36 | 20.3/17.8/4.8 | 76.2 | [36] |
| 3CzSF-BN/5TCzBN  /PhCzBCz | 519 | 37 | 23.1/20.8/21.3 | 82.9 |  |
| 5Cz-BO(host)/ 2tCz2CzBn | 468 | 64 | 21.21/13.67/4.57 | 34.33 | [37] |
| DOBN/mCP | 449 | 20 | 6.75/2.26/- | 2.77 | [38] |
| DOBN/mCP/TB-tCz | 447 | 20 | 27.70/16.68 | 9.81 |  |
| BN3/mCP | 550 | 45 | 18.5/9.0/- | 72.6 | [39] |
| BN3/mCP/  Al(MCzDBM)_3_ |  |  | 21.6/17.3/9.3 | 83.4 |  |
| BN3/mCP/  4CzIPN |  |  | 19.1/14.3/7.8 | 61.2 |  |
| H-FOBN | 460 | 32 | 9.2/-/- | 8.1 | [40] |
| Me-FOBN | 458 | 32 | 11.3/-/- | 9.3 |  |
| BN-R1/NPB/  DMFBD-TRZ | 610 | 44 | 20.7/16.0/6.3 | 33.5 | [41] |
| BN-R1/NPB/  DMFBD-TRZ/  PO-01 | 611 | 44 | 19.0/14.0/9.1 | 30 |  |
| BN-R2/NPB/  DMFBD-TRZ | 601 | 40 | 21.1/11.3/5.5 | 44.4 |  |
| BN-R2/NPB/  DMFBD-TRZ/  PO-01 | 600 | 41 | 20.4/14.3/9.9 | 41.8 |  |
| BN-R3/NPB/  DMFBD-TRZ | 585 | 43 | 19.9/15.1/10.1 | 57.1 |  |
| BN-R3/NPB/  DMFBD-TRZ/  PO-01 | 587 | 44 | 20.4/18.6/17. | 55.5 |  |
| PCzBN3  /mCP | 496 | 34 | 17.5 | 43.4 | [42] |
| PCzTBN5  /SimCP2 | 511 | 44 | 22.2/17.4/4.0 | 69.4 | [43] |
| PCzDBN3  /3Cz2BN | 479 | 28 | 17.9/8.2/- | 26.5 | [44] |
| PBO-F-10 | 474 | - | 15.0/9.9/3.5 | 30.7 | [45] |
| PAc-BSS | 458 | 31 | 13.1/-/- | 17.7 | [46] |
| P10/mCP | 505 | 40 | 19.4 | 60.2 | [47] |
| QAO-2/PVK/OXD-7 | 447 | 52 | 13.5 | 17 | [48] |
| QAO-3/PVK/OXD-7 | 447 | 52 | 10.0 | 8.8 |  |
| BN4/mCPCN | 510 | 49 | 20.6/20.5/10.7 | 66.3 | [49] |
| BN5/mCPCN | 506 | 48 | 26.5/17.6/11.1 | 79.37 |  |
| DCzBN–Au/DMIC-TRZ | 510 | 38 | 25.7/23.5/11.7 | 84.0 | [50] |
| BCzBN3tCz/Cy-mCP | 496 | 30 | 13.16/9.41 /  2.48 | 31.08 | [51] |
| BCzBN3tCz/Cy-mCP | 496 | 30 | 14.00/10.86/  3.17 | 34.05 |  |
| tBu3Cz-BN | 524 | 39 | 18.4/-/17.5 | 68.2 | [15] |
| PBN3DPOT97 | 491 | 39 | 12.7/12.2/11.9 | 23.9 | [52] |
| 2BN-ICz | 474 | 24 | 35.1/16.2/6.6 | 40.7 | [53] |
| D2-DBN | 471 | 17 | 35.3/-/29.5 | 42.6 | [54] |
| Endo-D2 | 472 | 38 | 22.6/17.1/- | - | [55] |
| DtCzBN-CNBT2 | 507 | 38 | 24.4/14.9/5.6 | - | [56] |
| DtCzBN-CNBT1 | 508 | 39 | 22.4/12.9/4.8 | - |  |
| 2GtBuCzCO2HDCzB | 495 | 27 | 27.9/27.6/22.3 | 61.2 | [57] |
| m-DT-BCzBN | 492 | 29 | 27.8/-/- | - | [58] |
| tBuCzBN-PXZ | 480 | 27 | 21.3% | 41.9 | [59] |
| tBuCzBN-PTZ | 478 | 25 | 19.0% | 30.4 |  |
| tBuCzBN-PSeZ | 474 | 29 | 15.2% | 20.3 |  |
| tBuCzBN-Ph-PXZ | 496 | 33 | 9.6% | 23.0 |  |
| tBuCzBN-Ph-PTZ | 496 | 36 | 11.6% | 30.1 |  |
| tBuCzBN-Ph-PSeZ | 496 | 36 | 19.4% | 50.5 |  |

**S13. Optimized atomic coordinates**

**(1) DMAC-XT**

| C | -5.10379 | -4.03115 | -1.4722 |
| --- | --- | --- | --- |
| C | -5.06419 | -3.61905 | -0.1408 |
| C | -4.84409 | -2.27395 | 0.15881 |
| C | -4.66649 | -1.30945 | -0.8425 |
| C | -4.70759 | -1.73935 | -2.1776 |
| C | -4.92109 | -3.08805 | -2.4833 |
| O | -4.43209 | -0.00625 | -0.5071 |
| C | -4.78859 | 0.93615 | -1.4293 |
| C | -4.83929 | 0.65285 | -2.8027 |
| C | -4.50919 | -0.73615 | -3.2839 |
| C | -5.08649 | 2.24355 | -1.0217 |
| C | -5.43489 | 3.24115 | -1.9333 |
| C | -5.48599 | 2.94355 | -3.2944 |
| C | -5.18329 | 1.65085 | -3.7214 |
| O | -4.13738 | -1.00754 | -4.40075 |
| C | -4.37338 | -5.57334 | 0.83617 |
| C | -4.62659 | -6.6091 | 1.74974 |
| C | -5.78266 | -6.50751 | 2.72004 |
| C | -6.57049 | -5.21717 | 2.6669 |
| C | -6.25106 | -4.22874 | 1.72204 |
| N | -5.22699 | -4.4604 | 0.79108 |
| C | -3.32538 | -5.70538 | -0.08045 |
| C | -2.50926 | -6.83814 | -0.06505 |
| C | -2.74621 | -7.85587 | 0.85616 |
| C | -3.80357 | -7.74557 | 1.7585 |
| C | -7.62902 | -5.00622 | 3.56346 |
| C | -8.372 | -3.82734 | 3.51051 |
| C | -8.06913 | -2.85681 | 2.55828 |
| C | -7.01826 | -3.06095 | 1.66202 |
| C | -5.22507 | -6.65744 | 4.12935 |
| C | -6.75803 | -7.60737 | 2.32195 |
| C | -4.79323 | 5.03536 | -0.66052 |
| C | -5.13253 | 6.25738 | -0.05794 |
| C | -6.45022 | 6.92801 | -0.37769 |
| C | -7.30694 | 6.2291 | -1.41019 |
| C | -6.89356 | 5.00798 | -1.96676 |
| N | -5.70301 | 4.40969 | -1.52663 |
| C | -3.58907 | 4.41111 | -0.3196 |
| C | -2.70921 | 5.01347 | 0.58174 |
| C | -3.03526 | 6.23691 | 1.16262 |
| C | -4.24442 | 6.8563 | 0.84846 |
| C | -8.52351 | 6.80065 | -1.8128 |
| C | -9.32818 | 6.15502 | -2.75103 |
| C | -8.92839 | 4.93262 | -3.2861 |
| C | -7.71988 | 4.35743 | -2.88867 |
| C | -6.14915 | 8.3299 | -0.8911 |
| C | -7.25418 | 6.90378 | 0.9156 |
| H | -5.27718 | -5.08821 | -1.72231 |
| H | -4.80892 | -1.96118 | 1.21281 |
| H | -4.94515 | -3.40876 | -3.53523 |
| H | -5.04441 | 2.4909 | 0.04931 |
| H | -5.76199 | 3.7197 | -4.02338 |
| H | -5.21537 | 1.41252 | -4.79479 |
| H | -3.14271 | -4.91091 | -0.819 |
| H | -1.6787 | -6.92699 | -0.78078 |
| H | -2.09977 | -8.74574 | 0.87176 |
| H | -3.9934 | -8.55333 | 2.48065 |
| H | -7.87395 | -5.77446 | 4.31168 |
| H | -9.19653 | -3.66395 | 4.22006 |
| H | -8.65826 | -1.929 | 2.51244 |
| H | -6.79204 | -2.29759 | 0.903 |
| H | -4.52435 | -5.81945 | 4.34275 |
| H | -6.06181 | -6.63663 | 4.86297 |
| H | -4.68183 | -7.62532 | 4.21219 |
| H | -6.87366 | -8.32451 | 3.16522 |
| H | -7.74663 | -7.15642 | 2.08094 |
| H | -6.3668 | -8.14469 | 1.4292 |
| H | -3.33326 | 3.43833 | -0.76488 |
| H | -1.75798 | 4.52167 | 0.83332 |
| H | -2.33948 | 6.71397 | 1.86853 |
| H | -4.5033 | 7.81901 | 1.31339 |
| H | -8.84404 | 7.76259 | -1.38629 |
| H | -10.2778 | 6.61064 | -3.06832 |
| H | -9.56522 | 4.42017 | -4.0222 |
| H | -7.41538 | 3.38533 | -3.30375 |
| H | -5.56184 | 8.26205 | -1.83409 |
| H | -7.10475 | 8.86564 | -1.08756 |
| H | -5.56114 | 8.88624 | -0.12723 |
| H | -7.46894 | 7.94684 | 1.23915 |
| H | -8.21251 | 6.36376 | 0.74601 |
| H | -6.66891 | 6.3833 | 1.70637 |

**(2) DMAC-XT-TCz**

| C | -0.94353 | 2.53637 | -4.90372 |
| --- | --- | --- | --- |
| C | -0.90243 | 2.96627 | -3.56862 |
| O | -0.66803 | 4.26947 | -3.23322 |
| C | -1.02453 | 5.21187 | -4.15542 |
| C | -1.07523 | 4.92857 | -5.52882 |
| C | -0.74513 | 3.53957 | -6.01002 |
| C | -1.15703 | 1.18767 | -5.20942 |
| C | -1.33973 | 0.24457 | -4.19832 |
| C | -1.30013 | 0.65667 | -2.86692 |
| C | -1.08003 | 2.00177 | -2.56732 |
| C | -1.32243 | 6.51927 | -3.74782 |
| C | -1.67083 | 7.51687 | -4.65942 |
| C | -1.72193 | 7.21927 | -6.02052 |
| C | -1.41923 | 5.92657 | -6.44752 |
| O | -0.37332 | 3.26818 | -7.12688 |
| N | -1.46293 | -0.18467 | -1.93504 |
| C | -2.12121 | 0.07742 | -0.75666 |
| C | -2.0866 | -1.09375 | -0.00083 |
| C | -1.3816 | -2.07755 | -0.76589 |
| C | -1.00471 | -1.48063 | -1.96829 |
| C | -1.0289 | -3.42511 | -0.54413 |
| C | -0.31662 | -4.12162 | -1.52736 |
| C | 0.0449 | -3.4963 | -2.71833 |
| C | -0.29233 | -2.16174 | -2.96273 |
| C | -2.73826 | 1.25154 | -0.30838 |
| C | -3.33893 | 1.22571 | 0.9538 |
| C | -3.32171 | 0.0717 | 1.73355 |
| C | -2.70003 | -1.09328 | 1.26925 |
| N | 0.002 | -5.33125 | -1.33236 |
| N | -3.87353 | 0.07651 | 2.87295 |
| C | -0.3287 | -6.36796 | -2.1729 |
| C | 0.20021 | -7.53078 | -1.61411 |
| C | 0.86505 | -7.16015 | -0.40134 |
| C | 0.72423 | -5.78099 | -0.25222 |
| C | -3.39789 | -0.60162 | 3.97046 |
| C | -4.27792 | -0.34545 | 5.0212 |
| C | -5.30675 | 0.51466 | 4.51929 |
| C | -5.02725 | 0.76056 | 3.17558 |
| C | 1.57565 | -7.87451 | 0.58571 |
| C | 2.11593 | -7.18711 | 1.67876 |
| C | 1.95769 | -5.80842 | 1.79839 |
| C | 1.25779 | -5.07709 | 0.83412 |
| C | -1.04889 | -6.36296 | -3.37353 |
| C | -1.2334 | -7.58737 | -4.02265 |
| C | -0.71777 | -8.76683 | -3.49062 |
| C | -0.00011 | -8.75301 | -2.28896 |
| C | -6.45339 | 1.10785 | 5.08748 |
| C | -7.27309 | 1.92023 | 4.29544 |
| C | -6.96598 | 2.14627 | 2.95577 |
| C | -5.8356 | 1.57017 | 2.3685 |
| C | -2.26613 | -1.41397 | 4.10984 |
| C | -2.02782 | -1.98206 | 5.36485 |
| C | -2.88772 | -1.74578 | 6.43479 |
| C | -4.01473 | -0.93076 | 6.2772 |
| C | 2.87091 | -7.94072 | 2.72905 |
| C | 3.35238 | -6.97296 | 3.80197 |
| C | 1.95758 | -8.9835 | 3.35989 |
| C | 4.07027 | -8.63205 | 2.09412 |
| C | -0.93315 | -10.0614 | -4.21077 |
| C | -1.73583 | -9.80955 | -5.48033 |
| C | 0.41495 | -10.669 | -4.57552 |
| C | -1.6973 | -11.0224 | -3.30956 |
| C | -8.49122 | 2.55206 | 4.89375 |
| C | -9.20661 | 3.3803 | 3.83462 |
| C | -9.42726 | 1.4647 | 5.40463 |
| C | -8.07927 | 3.45352 | 6.05012 |
| C | -2.60393 | -2.36936 | 7.76581 |
| C | -1.33212 | -3.20243 | 7.67614 |
| C | -2.42079 | -1.27537 | 8.80945 |
| C | -3.76956 | -3.26426 | 8.1658 |
| C | -3.54288 | 10.5048 | -4.13631 |
| C | -2.68616 | 11.2037 | -3.10382 |
| C | -1.36847 | 10.5331 | -2.78407 |
| C | -1.02917 | 9.31109 | -3.38664 |
| N | -1.93895 | 8.68542 | -4.25276 |
| C | -3.1295 | 9.28371 | -4.69289 |
| C | -0.48036 | 11.132 | -1.87767 |
| C | 0.7288 | 10.5126 | -1.5635 |
| C | 1.05485 | 9.2892 | -2.14438 |
| C | 0.17499 | 8.68683 | -3.04573 |
| C | -3.95582 | 8.63316 | -5.61479 |
| C | -5.16433 | 9.20834 | -6.01222 |
| C | -5.56412 | 10.4307 | -5.47716 |
| C | -4.75945 | 11.0764 | -4.53892 |
| C | -2.3851 | 12.6056 | -3.61723 |
| C | -3.49012 | 11.1795 | -1.81053 |
| H | -1.18109 | 0.86696 | -6.26136 |
| H | -1.51312 | -0.81249 | -4.44843 |
| H | -1.04486 | 2.31455 | -1.51331 |
| H | -1.28036 | 6.76662 | -2.67682 |
| H | -1.99793 | 7.99542 | -6.74951 |
| H | -1.45131 | 5.68824 | -7.52091 |
| H | -1.3114 | -3.92479 | 0.39423 |
| H | 0.60444 | -4.06131 | -3.47839 |
| H | -0.00832 | -1.66487 | -3.90212 |
| H | -2.75 | 2.16119 | -0.92676 |
| H | -3.83281 | 2.13081 | 1.33703 |
| H | -2.68997 | -2.00132 | 1.89002 |
| H | 1.70422 | -8.96337 | 0.49715 |
| H | 2.39012 | -5.28695 | 2.66503 |
| H | 1.1309 | -3.98827 | 0.92557 |
| H | -1.45476 | -5.42965 | -3.79091 |
| H | -1.7945 | -7.61977 | -4.96823 |
| H | 0.40447 | -9.68802 | -1.87416 |
| H | -6.70152 | 0.93338 | 6.14483 |
| H | -7.62305 | 2.78804 | 2.35046 |
| H | -5.59022 | 1.74655 | 1.31082 |
| H | -1.5891 | -1.59851 | 3.26274 |
| H | -1.14755 | -2.62563 | 5.50955 |
| H | -4.68985 | -0.74817 | 7.12624 |
| H | 3.91369 | -7.53326 | 4.58284 |
| H | 4.01983 | -6.2109 | 3.34095 |
| H | 2.47589 | -6.46774 | 4.26597 |
| H | 2.51797 | -9.54442 | 4.14098 |
| H | 1.60624 | -9.69111 | 2.57592 |
| H | 1.0815 | -8.47728 | 3.82356 |
| H | 4.09362 | -8.40882 | 1.00399 |
| H | 5.00602 | -8.26044 | 2.56849 |
| H | 3.98768 | -9.73156 | 2.2458 |
| H | -1.89596 | -10.7721 | -6.01575 |
| H | -2.72101 | -9.36555 | -5.21377 |
| H | -1.17739 | -9.10729 | -6.13893 |
| H | 0.25594 | -11.6316 | -5.11104 |
| H | 1.00129 | -10.8537 | -3.64769 |
| H | 0.97253 | -9.966 | -5.23407 |
| H | -1.90963 | -10.5284 | -2.33504 |
| H | -2.65642 | -11.3052 | -3.7983 |
| H | -1.08457 | -11.9353 | -3.13682 |
| H | -10.1123 | 3.85005 | 4.27945 |
| H | -8.52255 | 4.17494 | 3.46127 |
| H | -9.50766 | 2.72152 | 2.98955 |
| H | -10.3333 | 1.93343 | 5.84971 |
| H | -8.90467 | 0.85983 | 6.1791 |
| H | -9.72768 | 0.80654 | 4.55886 |
| H | -6.97304 | 3.42322 | 6.16897 |
| H | -8.40065 | 4.49786 | 5.8384 |
| H | -8.56281 | 3.09842 | 6.98759 |
| H | -1.12112 | -3.66605 | 8.66573 |
| H | -1.46596 | -4.00191 | 6.91346 |
| H | -0.48028 | -2.54845 | 7.38383 |
| H | -2.20972 | -1.73796 | 9.79951 |
| H | -3.35045 | -0.66697 | 8.87544 |
| H | -1.56879 | -0.62201 | 8.51621 |
| H | -4.54861 | -3.23689 | 7.37138 |
| H | -3.40761 | -4.30894 | 8.29389 |
| H | -4.20408 | -2.90111 | 9.12397 |
| H | -0.73925 | 12.0947 | -1.41274 |
| H | 1.42457 | 10.9897 | -0.8576 |
| H | 2.00608 | 8.79739 | -1.8928 |
| H | 0.4308 | 7.71406 | -3.49101 |
| H | -3.65132 | 7.66106 | -6.02988 |
| H | -5.80117 | 8.6959 | -6.74832 |
| H | -6.51372 | 10.8864 | -5.79444 |
| H | -5.07999 | 12.0383 | -4.11241 |
| H | -1.79778 | 12.5378 | -4.56022 |
| H | -3.34069 | 13.1414 | -3.81369 |
| H | -1.79709 | 13.162 | -2.85335 |
| H | -3.70488 | 12.2226 | -1.48698 |
| H | -4.44845 | 10.6395 | -1.98012 |
| H | -2.90485 | 10.659 | -1.01976 |

**(3) TCz-XT**

| C | 0.33662 | -4.57924 | -3.45127 |
| --- | --- | --- | --- |
| C | 0.37622 | -4.16714 | -2.11987 |
| C | 0.59632 | -2.82204 | -1.82027 |
| C | 0.77392 | -1.85754 | -2.82157 |
| C | 0.73282 | -2.28744 | -4.15667 |
| C | 0.51932 | -3.63614 | -4.46237 |
| O | 1.00832 | -0.55434 | -2.48617 |
| C | 0.65182 | 0.38806 | -3.40837 |
| C | 0.60112 | 0.10476 | -4.78177 |
| C | 0.93122 | -1.28424 | -5.26297 |
| C | 0.35392 | 1.69546 | -3.00077 |
| C | 0.00552 | 2.69306 | -3.91237 |
| C | -0.04558 | 2.39546 | -5.27347 |
| C | 0.25712 | 1.10276 | -5.70047 |
| O | 1.30303 | -1.55563 | -6.37983 |
| C | -0.44486 | -4.74639 | -0.0096 |
| C | -0.41025 | -5.91756 | 0.74622 |
| C | 0.29474 | -6.90136 | -0.01884 |
| C | 0.67164 | -6.30445 | -1.22124 |
| N | 0.21342 | -5.00848 | -1.18799 |
| C | -1.06191 | -3.57228 | 0.43867 |
| C | -1.66258 | -3.59811 | 1.70085 |
| C | -1.64536 | -4.75212 | 2.4806 |
| C | -1.02368 | -5.9171 | 2.0163 |
| C | 0.64745 | -8.24893 | 0.20293 |
| C | 1.35973 | -8.94543 | -0.78031 |
| C | 1.72125 | -8.32012 | -1.97128 |
| C | 1.38402 | -6.98555 | -2.21568 |
| N | -2.19718 | -4.7473 | 3.62 |
| N | 1.67835 | -10.1551 | -0.58531 |
| C | -1.72154 | -5.42543 | 4.71751 |
| C | -2.60157 | -5.16927 | 5.76825 |
| C | -3.6304 | -4.30916 | 5.26634 |
| C | -3.3509 | -4.06326 | 3.92263 |
| C | 1.34765 | -11.1918 | -1.42585 |
| C | 1.87656 | -12.3546 | -0.86706 |
| C | 2.5414 | -11.984 | 0.34572 |
| C | 2.40058 | -10.6048 | 0.49483 |
| C | -0.58978 | -6.23779 | 4.85689 |
| C | -0.35147 | -6.80588 | 6.1119 |
| C | -1.21138 | -6.5696 | 7.18185 |
| C | -2.33838 | -5.75458 | 7.02425 |
| C | -4.77704 | -3.71597 | 5.83453 |
| C | -5.59674 | -2.90358 | 5.04249 |
| C | -5.28963 | -2.67754 | 3.70282 |
| C | -4.15925 | -3.25364 | 3.11555 |
| C | 0.62746 | -11.1868 | -2.62648 |
| C | 0.44295 | -12.4112 | -3.2756 |
| C | 0.95858 | -13.5906 | -2.74357 |
| C | 1.67624 | -13.5768 | -1.54191 |
| C | 3.252 | -12.6983 | 1.33276 |
| C | 3.79228 | -12.0109 | 2.42582 |
| C | 3.63403 | -10.6322 | 2.54544 |
| C | 2.93414 | -9.90091 | 1.58117 |
| C | -0.92758 | -7.19318 | 8.51287 |
| C | -6.81487 | -2.27176 | 5.6408 |
| C | 0.7432 | -14.8853 | -3.46372 |
| C | 4.54726 | -12.7645 | 3.4761 |
| C | 5.02872 | -11.7968 | 4.54902 |
| C | 3.63393 | -13.8073 | 4.10694 |
| C | 5.74661 | -13.4559 | 2.84117 |
| C | -0.05948 | -14.6334 | -4.73328 |
| C | 2.0913 | -15.4928 | -3.82847 |
| C | -0.02095 | -15.8462 | -2.56251 |
| C | -7.53026 | -1.44351 | 4.58167 |
| C | 0.34423 | -8.02624 | 8.42319 |
| C | -0.74444 | -6.09918 | 9.5565 |
| C | -2.09321 | -8.08807 | 8.91285 |
| C | -7.75091 | -3.35912 | 6.15168 |
| C | -6.40292 | -1.3703 | 6.79717 |
| C | -1.22117 | 4.67713 | -4.05952 |
| C | -1.21038 | 5.86896 | -3.33596 |
| C | -0.20789 | 5.74578 | -2.32105 |
| C | 0.36647 | 4.48203 | -2.45218 |
| N | -0.2626 | 3.8616 | -3.50571 |
| C | -2.08783 | 4.45085 | -5.13559 |
| C | -2.9715 | 5.47741 | -5.48181 |
| C | -2.98569 | 6.67989 | -4.77904 |
| C | -2.11191 | 6.88874 | -3.70567 |
| C | 0.26434 | 6.59679 | -1.30012 |
| C | 1.28738 | 6.15492 | -0.45316 |
| C | 1.84021 | 4.88615 | -0.61046 |
| C | 1.39022 | 4.02348 | -1.61436 |
| N | -3.79652 | 7.59255 | -5.11425 |
| N | 1.71421 | 6.91556 | 0.46444 |
| C | -3.73311 | 8.2942 | -6.29506 |
| C | -4.79212 | 9.20116 | -6.28803 |
| C | -5.50132 | 9.02324 | -5.05694 |
| C | -4.8563 | 8.01242 | -4.34538 |
| C | 0.92468 | 7.77226 | 1.19474 |
| C | 1.7608 | 8.42792 | 2.09759 |
| C | 3.08904 | 7.93625 | 1.88731 |
| C | 3.02823 | 6.99358 | 0.86173 |
| C | -2.82685 | 8.1945 | -7.3576 |
| C | -3.0107 | 9.05055 | -8.44752 |
| C | -4.0587 | 9.96773 | -8.4712 |
| C | -4.95539 | 10.05356 | -7.39985 |
| C | -6.63633 | 9.63191 | -4.48178 |
| C | -7.08155 | 9.20949 | -3.22379 |
| C | -6.41474 | 8.19659 | -2.53858 |
| C | -5.28745 | 7.57721 | -3.08642 |
| C | -0.45275 | 8.01293 | 1.12415 |
| C | -0.99121 | 8.95479 | 2.00593 |
| C | -0.18298 | 9.62692 | 2.91976 |
| C | 1.19247 | 9.37359 | 2.97629 |
| C | 4.34079 | 8.20825 | 2.47778 |
| C | 5.47856 | 7.53132 | 2.02339 |
| C | 5.38408 | 6.59486 | 0.99663 |
| C | 4.15554 | 6.30707 | 0.39464 |
| C | -4.22762 | 10.87018 | -9.6536 |
| C | -8.28564 | 9.85235 | -2.60904 |
| C | -0.79108 | 10.62967 | 3.8502 |
| C | 6.80939 | 7.81648 | 2.64676 |
| C | 7.87696 | 6.95815 | 1.98111 |
| C | 7.15233 | 9.28839 | 2.45867 |
| C | 6.75284 | 7.49315 | 4.13397 |
| C | -2.28902 | 10.72102 | 3.5907 |
| C | -0.55161 | 10.19549 | 5.29022 |
| C | -0.15157 | 11.99198 | 3.61639 |
| C | -8.56181 | 9.2204 | -1.25113 |
| C | -8.02943 | 11.34313 | -2.43171 |
| C | -9.49008 | 9.64763 | -3.51839 |
| C | -3.13395 | 10.58188 | -10.6736 |
| C | -5.59014 | 10.62455 | -10.2882 |
| C | -4.13069 | 12.32151 | -9.20219 |
| H | 0.16323 | -5.6363 | -3.70138 |
| H | 0.63149 | -2.50927 | -0.76626 |
| H | 0.49526 | -3.95685 | -5.51431 |
| H | 0.39599 | 1.94281 | -1.92977 |
| H | -0.32158 | 3.17161 | -6.00246 |
| H | 0.22503 | 0.86443 | -6.77386 |
| H | -1.07366 | -2.66262 | -0.1797 |
| H | -2.15646 | -2.693 | 2.08408 |
| H | -1.01362 | -6.82514 | 2.63708 |
| H | 0.36495 | -8.74861 | 1.14128 |
| H | 2.28079 | -8.88512 | -2.73134 |
| H | 1.66803 | -6.48869 | -3.15507 |
| H | 0.08725 | -6.42233 | 4.00979 |
| H | 0.5288 | -7.44945 | 6.25661 |
| H | -3.0135 | -5.57199 | 7.87329 |
| H | -5.02517 | -3.89043 | 6.89189 |
| H | -5.9467 | -2.03578 | 3.09751 |
| H | -3.91387 | -3.07726 | 2.05787 |
| H | 0.22159 | -10.2535 | -3.04386 |
| H | -0.11815 | -12.4436 | -4.22118 |
| H | 2.08082 | -14.5118 | -1.12711 |
| H | 3.38057 | -13.7872 | 1.2442 |
| H | 4.06647 | -10.1108 | 3.41208 |
| H | 2.80725 | -8.81209 | 1.67263 |
| H | 5.59004 | -12.3571 | 5.32989 |
| H | 5.69618 | -11.0347 | 4.088 |
| H | 4.15224 | -11.2916 | 5.01302 |
| H | 4.19432 | -14.3682 | 4.88803 |
| H | 3.28259 | -14.5149 | 3.32297 |
| H | 2.75785 | -13.3011 | 4.57061 |
| H | 5.76997 | -13.2326 | 1.75104 |
| H | 6.68237 | -13.0843 | 3.31554 |
| H | 5.66403 | -14.5554 | 2.99285 |
| H | -0.21961 | -15.5959 | -5.2687 |
| H | -1.04466 | -14.1894 | -4.46672 |
| H | 0.49896 | -13.9311 | -5.39188 |
| H | 1.93229 | -16.4555 | -4.36399 |
| H | 2.67764 | -15.6775 | -2.90064 |
| H | 2.64888 | -14.7898 | -4.48702 |
| H | -0.23328 | -15.3522 | -1.58799 |
| H | -0.98007 | -16.129 | -3.05125 |
| H | 0.59178 | -16.7592 | -2.38977 |
| H | -8.43592 | -0.97376 | 5.02651 |
| H | -6.8462 | -0.64888 | 4.20832 |
| H | -7.83131 | -2.10229 | 3.7366 |
| H | 0.55523 | -8.48987 | 9.41278 |
| H | 0.21039 | -8.82573 | 7.66051 |
| H | 1.19607 | -7.37226 | 8.13088 |
| H | -0.53337 | -6.56178 | 10.54656 |
| H | -1.6741 | -5.49078 | 9.6225 |
| H | 0.10756 | -5.44582 | 9.26326 |
| H | -2.87226 | -8.06071 | 8.11843 |
| H | -1.73126 | -9.13275 | 9.04094 |
| H | -2.52773 | -7.72493 | 9.87102 |
| H | -8.65698 | -2.89039 | 6.59676 |
| H | -7.22832 | -3.96399 | 6.92615 |
| H | -8.05133 | -4.01728 | 5.30591 |
| H | -5.2967 | -1.40059 | 6.91602 |
| H | -6.7243 | -0.32595 | 6.58545 |
| H | -6.88646 | -1.72539 | 7.73464 |
| H | -2.07397 | 3.49999 | -5.68847 |
| H | -3.66635 | 5.33323 | -6.32228 |
| H | -2.1282 | 7.84072 | -3.1548 |
| H | -0.16801 | 7.59992 | -1.17054 |
| H | 2.64333 | 4.55784 | 0.06571 |
| H | 1.82466 | 3.02092 | -1.74138 |
| H | -2.00028 | 7.46903 | -7.33564 |
| H | -2.31695 | 8.99897 | -9.2996 |
| H | -5.78093 | 10.7801 | -7.42481 |
| H | -7.16747 | 10.43234 | -5.0177 |
| H | -6.78078 | 7.87907 | -1.55107 |
| H | -4.75848 | 6.77707 | -2.54795 |
| H | -1.08816 | 7.48036 | 0.40123 |
| H | -2.0699 | 9.16836 | 1.9776 |
| H | 1.8252 | 9.90784 | 3.70033 |
| H | 4.42222 | 8.94662 | 3.28906 |
| H | 6.29017 | 6.07364 | 0.65411 |
| H | 4.07743 | 5.56817 | -0.41649 |
| H | 8.86641 | 7.17016 | 2.44457 |
| H | 7.62634 | 5.88248 | 2.11856 |
| H | 7.91829 | 7.19444 | 0.89426 |
| H | 8.1417 | 9.50148 | 2.92182 |
| H | 6.3725 | 9.91564 | 2.94568 |
| H | 7.1934 | 9.5236 | 1.37158 |
| H | 5.73605 | 7.12361 | 4.39544 |
| H | 7.50483 | 6.70765 | 4.37117 |
| H | 6.97619 | 8.41145 | 4.72186 |
| H | -2.74114 | 11.46655 | 4.28247 |
| H | -2.46402 | 11.03831 | 2.53833 |
| H | -2.75637 | 9.72545 | 3.76156 |
| H | -1.00338 | 10.94041 | 5.98288 |
| H | 0.54308 | 10.12935 | 5.48005 |
| H | -1.01932 | 9.1999 | 5.46002 |
| H | 0.61212 | 11.91058 | 2.81083 |
| H | -0.93494 | 12.72161 | 3.31185 |
| H | 0.33522 | 12.33915 | 4.55515 |
| H | -9.45703 | 9.69836 | -0.79407 |
| H | -8.74904 | 8.13095 | -1.38072 |
| H | -7.6816 | 9.37001 | -0.58658 |
| H | -8.92413 | 11.82209 | -1.97468 |
| H | -7.82825 | 11.80507 | -3.42414 |
| H | -7.14911 | 11.49162 | -1.76706 |
| H | -9.18209 | 9.07001 | -4.41854 |
| H | -10.2778 | 9.08515 | -2.96892 |
| H | -9.89039 | 10.63763 | -3.83212 |
| H | -3.25954 | 11.25284 | -11.5526 |
| H | -2.13823 | 10.76139 | -10.2098 |
| H | -3.20478 | 9.52126 | -11.0034 |
| H | -5.71676 | 11.29507 | -11.1675 |
| H | -6.38941 | 10.83585 | -9.54305 |
| H | -5.65993 | 9.56374 | -10.6177 |
| H | -3.95789 | 12.35805 | -8.10329 |
| H | -3.28348 | 12.81695 | -9.7271 |
| H | -5.07941 | 12.84989 | -9.44615 |

**S14. Reference**

[1] Y. Liu, Y. Wang, C. Li, Z. Ren, D. Ma, S. Yan, *Macromolecules,* **2018**, 51, 4615.

[2] W. L. Tsai, M. H. Huang, W. K. Lee, Y. J. Hsu, K. C. Pan, Y. H. Huang, H. C. Ting, M. Sarm, Y. Y. Ho, H. C. Hu, C. C. Chen, M. T. Lee, K. T. Wong, C. C. Wu, *Chem. Commun.,* **2015**, 51, 13662.

[3] Y. Liu, G. Xie, Z. Ren, S. Yan, *ACS Appl. Polym. Mater*., **2019**, 8, 2204.

[4] Y. Liu, L. Hua, Z. Zhao, S. Ying, Z. Ren, S. Yan, *Adv. Sci.,* **2021**, 8, 2101326.

[5] J. Ding, J. Lü, Y. Cheng, Z. Xie, L., Jing, X. Wang, F. Wang, *Adv. Funct. Mater*., **2008**, 18, 2754.

[6] L. Yang, P. Wang, K. Zhang, S. Wang, S. Shao, L. Wang. *Dyes Pigm*., **2023**, 216, 111371.

[7] X. Ban, Y. Liu, J. Pan, F. Chen, A. Zhu, W. Jiang, Y. Sun, Y. Dong. *Org. Electron*., **2020**, 80, 105601.

[8] C. Li, A. K. Harrison, Y. Liu, Z. Zhao, C. Zeng, F. B. Dias, Z. Ren, S. Yan, M. R. Bryce. *Angew. Chem. Int. Ed.*, **2022**, 61, e202115140.

[9] K. Albrecht, K. Matsuoka, K. Fujita, K. Yamamoto. *Mater. Chem. Front*., **2018**, 2, 1097.

[10] D. Sun, E. Duda, X. Fan, R. Saxena, M. Zhang, S. Bagnich, X. Zhang, A. Köhler, E. Zysman-Colman, *Adv. Mater.,* **2022**, *34*, 2110344.

[11] G. Zhao, D. Liu, P. Wang, X. Huang, H. Chen, Y. Zhang, D. Zhang, W. Jiang, Y. Sun, L. Duan, *Angew. Chem. Int. Ed*., **2022**, 61, e202212861.

[12] W. Zhang, X. Qian, W. Shi, J. Jiang, H. Zhou, Z. Wu, Z. Yang, X. Ban, Y. Xin, *Opt. Mater.*, **2025**, 167, 117163.

[13] W. Zhang, J. Yu, Q. Cao, Y. Qian, J. Wang, C. Yang, H. Zhuang, W. Bian, Y. Xin, X. Ban, *J. Mater. Chem. C*, **2023**, 11, 16247-16257.

[14] J. Fan, Y. Lou, G. Zhao, T. Zhou, H. Chen, R. Ji, W. Tian, W. Jiang, Y. Sun, Y. Qian, *Dyes and Pigments Dyes Pigm.*, **2025**, 238, 112723.

[15] K. Shi, Y. Xie, L. Hua, S. Li, Z. Yang, Y. Yin, Z. Wang, S. Ying, Y. Liu, Z. Ren, S. Yan, *ACS Mater. Lett*., **2024**, 6, 1491–1503.

[16] C. Si, D. Sun, T. Matulaitis, D. B. Cordes, A. M. Z. Slawin, E. Zysman-Colman, *Sci. China Chem.*, **2024**, 67, 1613–1623.

[17] Z. Ma, Y. Wan, W. Dong, Z. Si, Q. Duana, S. Shao, *Chin. Chem. Lett*., **2021**, 32, 703–707.

[18] J. Wu, X. Wang, W. Tian, H. Liu, C. Ge, Z. Zhang, Y. Song, Z. Cai, K. Sun, W. Jiang, *J. Ind. Eng. Chem*., **2025**, 143, 165–175.

[19] N. Ikeda, S. Oda, R. Matsumoto, M. Yoshioka, D. Fukushima, K. Yoshiura, N. Yasuda, T. Hatakeyama. *Adv. Mater.,* **2020**, 32, 2004072.

[20] H. J. Kim, M. Godumala, S. K. Kim, J. Yoon, C. Y. Kim, H. Park, J. H. Kwon, M. J. Cho, D. H. Choi. *Adv. Opt. Mater*., **2020**, 8, 1902175.

[21] S. Oda, B. Kawakami, Y. Yamasaki, R. Matsumoto, M. Yoshiok, D. Fukushima, S. Nakatsuka, T. Hatakeyama. *J. Am. Chem. Soc.* **2021**, 144, 106-112.

[22] H. J. Kim, H. Kang, J. E. Jeong, S. H. Park, C. W. Koh, C. W. Kim, H. Y. Woo, M. J. Cho, S. Park, D. H. Choil. *Adv. Funct. Mater.,* **2021**, 31, 2102588.

[23] J. Hwang, H. Kang, J. E. Jeong, H. Y. Woo, M. J. Cho, S. Park, D. H. Choi. *Chem. Eng. J*., **2021**, 416, 129185.

[24] S. Xu, Q. Yang, Y. Zhang, H. Li, Q. Xue, G. Xie, M. Gu, J. Jin, L. Huang, R. Chen, *Chin. Chem. Lett*., **2021**, 32, 1372-1376.

[25] R. Ma, Z. Ma, X. Wang, Z. Si, Q. Duan, S. Shao, *Chem. Eng. J*., **2022**, 447, 137517.

[26] T. Xu, X. Liang, G. Xie, *Front. Chem*., **2021**, 9, 691172.

[27] D. Zhou, S. Wu, G. Cheng, C. Che, *J. Mater. Chem. C*, **2022**, 10, 4590-4596.

[28] F. Xie, H. Li, K. Zhang, H. Wang, Y. Li, J. Tang. *ACS Appl. Mater*., **2023**, 15, 39669-39676.

[29] B. Du, K. Zhang, P. Wang, X. Wang, S. Wang, S. Shao, L. Wang, *J. Mater. Chem. C*, **2023**, 11, 9578-9585.

[30] X. Cai, Y. Xu, Y. Pan, L. Li, Y. Pu, X. Zhuang, C. Li, Y. Wang. *Angew. Chem. Int. Ed*., **2023**, 62, e202216473.

[31] H. Wang, X. Fan, J. Chen, Y. Cheng, X. Zhang, H. Wu, X. Xiong, J. Yu, K. Wang, X. Zhang. *Adv. Funct. Mater.,* **2023**, 33, 2306394.

[32] T. Wang, X. Yin, X. Cao, C. Yang. *Angew. Chem. Int. Ed.,* **2023**, 135, e202301988.

[33] K. Zhang, X. Wang, Y. Chang, Y. Wu, S. Wang, L. Wang, *Angew. Chem. Int. Ed.,* **2023**, 62, e202313084.

[34] X. Zhuang, J. Liang, X. Song, Q. Wang, H. Bi, B. Liang, Y. Wang. *Chem. Eng. J.,* **2024**, 493, 152857.

[35] F. Huang, Y. Cheng, H. Wu, X. Xiong, J. Yu, X. Fan, K. Wang, X. Zhang. *Chem. Eng. J.,* **2023**, 465, 142900.

[36] X. Zhuang, B. Liang, C. Jiang, S. Wang, H. Bi, Y. Wang. *Adv. Opt. Mater*., **2024**, 12, 2400490.

[37] H. Wang, F. Xie, H. Li, K. Zhang, Y. Zhu, H. Shi, Y. Li, J. Tang. *Adv. Opt. Mater.,* **2024**, 12, 2400025.

[38] J. Jin, M. Chen, H. Jiang, B. Zhang, Z. Xie, W. Y. Wong. *ACS Mater. Lett.*, **2024**, 6*,* 3246-3253.

[39] K. Hoshi, H. Sasabe, Y. Chiba, N. Yoshida, T. Nakamura, K. Nagasawa, Y. Sayama, H. Katagiri, J. Kido. *Adv. Opt. Mater.,* **2024**, 12, 2303303.

[40] X. Song, S. Shen, B. He, S. Zou, A. A. Vaitusionak, S. V. Kostjuk, Y. Wang, Y. Wang, Y. Zhang. *Adv. Opt. Mater.,* **2024**, 12, 2401505.

[41] X. Cai, Y. Pan, X. Song, C. Li, Y. Pu, X. Zhuang, H. Bi, Y. Wang. *Adv. Opt. Mater.,* **2024**, 12, 2302811.

[42] T. Wang, Y. Zou, Z. Huang, N. Li, J. Miao, C. Yang. *Angew. Chem. Int. Ed*., **2022**, 61, e202211172.

[43] T. Wang, Z. Huang, H. Zhang, J. Miao, C. Yang. *Adv. Funct. Mater.*, **2024**, 34, 2408119.

[44] W. Luo, T. Wang, Z Huang, H. Huang, N. Li, C. Yang, *Adv. Funct. Mater.,* **2024**, 34, 2310042.

[45] F. Chen, J. Hu, X. Wang, S. Shao, L. Wang, X. Jing, F. Wang. *Sci. China Chem.,* **2020**, 63, 1112-1120.

[46] F. Chen, L. Zhao, X. Wang, Q. Yang, W. Li, H. Tian, S. Shao, L. Wang, X. Jing, F. Wang. *Sci. China Chem.,* **2021**, 64, 547-551.

[47] K. Xu, N. Li, H. Lin, Z. Ye, P. Jiang, J. Miao, X. Cao, C. Yang. *Chem. Comm.,* **2024**, 60, 10318-10321.

[48] W. Zhang, H. Zhuang, S. Chen, S. Hu, Y. Qian, Z. Wu, Z. Yang, J. Chen, Y. Xin, X. Ban. *Chem. Eng. J.*, **2024**, 498, 155350.

[49] X. Wu, W. Huang, B. Su, S. Wang, L. Yuan, W. Zheng, H. Zhang, Y. Zheng, W. Zhu, P. Chou. *Adv. Mater*., **2022**, 34, 2105080.

[50] J. Wang, N. Li, C. Zhong, J. Miao, Z. Huang, M. Yu, Y. Hu, S. Luo, Y. Zou, K. Li, C. Yang. *Adv. Mater*., **2023**, 35, 2208378.

[51] S. H. Park, N. Y. Kwon, C. W. Koh, J. Y. Park, M. J. Kang, H. Kwak, C. Y. Park, S. Park, M. J. Cho, D. H. Choi. *Chem. Eng. J.,* **2024**, 481, 148484.

[52] R. Tian, Z. Yang, Z. Wang, J. Dong, W. Li, G. Li, H. Xu, *J. Mater. Chem C,* **2025**. DOI: 10.1039/D5TC02304A.

[53] Y. He, J. Liu, Z. Zhang, G. Chen, Y. Wang, G. Yuan, F. Xie, J. Tang, Y. Li. *Matter*, **2025**. 102188.

[54] K. Zhang, X. Wang, M. Wang, S. Wang. *Angew. Chem. Int. Ed.,* **2025**, 137, e202423812.

[55] Y. Chang, K. Zhang, L. Zhao, X. Wang, S. Wang, S. Shao, L. Wang. *Angew. Chem. Int. Ed.,* **2025**, 64, e202415607.

[56] D. Chen, H. Wang, D. Sun, S. Wu, K. Wang, X. Zhang, E. Z. Colman. *Adv. Mater.,* **2024**, 36, 2412761.

[57] S. Wu, D. Chen, X. Zhang, D. Sun, E. Z. Colman. *Adv. Mater.,* **2025**, 37, 2415289.

[58] Z. Yang, S. Li, L. Hua, S. Ying, Y. Liu, Z. Ren, S. Yan. *Chem. Sci*., **2025**, 16, 3904-3915.

[59] S. Li, Z. Yang, Y. Xie, L. Hua, S. Ying, Y. Liu, Z. Ren, S. Yan. *Chem. Sci.*, **2024**, 15, 18335-18346.
